# Deciphering the Multifaceted Immune Landscape of Unresectable Primary Liver Cancer to Predict Immunotherapy Response

**DOI:** 10.1002/advs.202309631

**Published:** 2024-10-28

**Authors:** Jun Xue, Shuai Yang, Si‐Si Zhang, Jun Fan, Zi‐Long Wu, Cheng‐Jun Sui, Yong‐Qiang Yang, Jin‐Feng Zhang, Pian Liu, De‐Jun Zhang, Xin‐Yao Qiu, Tao Zhang, Lei Chen, Gang Wu, Hong‐Yang Wang, Jing Tang

**Affiliations:** ^1^ Cancer Center Union Hospital Tongji Medical College Huazhong University of Science and Technology Wuhan 430022 China; ^2^ Institute of Radiation Oncology Union Hospital Tongji Medical College Huazhong University of Science and Technology Wuhan 430022 China; ^3^ Fudan University Shanghai Cancer Center Department of Oncology Shanghai Medical College Fudan University Shanghai 200032 China; ^4^ Hubei Key Laboratory of Precision Radiation Oncology Wuhan 430022 China; ^5^ Department of Pathology Union Hospital Tongji Medical College Huazhong University of Science and Technology Wuhan 430022 China; ^6^ The International Cooperation Laboratory on Signal Transduction Eastern Hepatobiliary Surgery Hospital Naval Medical University Shanghai 200438 China; ^7^ National Center for Liver Cancer Shanghai 200441 China

**Keywords:** efficacy prediction model, immune‐checkpoint inhibition‐based therapy, peripheral immune landscapes, primary liver cancer

## Abstract

Immunotherapies employing PD‐1/PD‐L1 immune checkpoint inhibitors (ICIs) are vital for primary liver cancer (PLC), but response rates remain unsatisfying. Accurate differentiation of responders from non‐responders to immunotherapy is imperative. Here, single‐cell‐scaled mass cytometry analysis on sequential peripheral blood mononuclear cells (PBMCs) from ICI‐treated PLC patients is conducted, and tissue residence of immune subpopulations is assessed via multiplex immunohistochemistry. In the discovery cohort (n = 24), responders have lower baseline B cell and HLA‐DR^+^CD8^+^T cell, and higher CD14^+^CD16^−^ classical monocyte (CM) proportions. CMs decrease more in responders PBMCs, while HLA‐DR^+^CD8^+^T cells conformably amplify after ICI‐exposure. Responsive individuals display upregulated exhaustion and activation markers in peripheral immune lineages. In the expanded cohort of 77 patients, the augment of the B cells in non‐responders is re‐confirmed. Responders demonstrate much higher enrichment of B cells or tertiary lymphoid structures in tumor compared to non‐responders. A prospective model that excelled in early discrimination of responders is developed using generalized linear model and achieves a satisfactory AUC over 0.9 in all three independent cohorts. Integratedly, the study unveils dynamic immune landscapes in PLC patients undergoing ICI‐based therapy, aiding in PLC patient stratification for ICI‐based treatment and fostering new response monitoring strategies.

## Graphical abstract

Predictive biomarkers for efficacy of ICI‐based therapy in primary liver cancer (PLC) remain ambiguous. Here, sequential circulating and in situ immune characteristics are portrayed by single‐cell‐scaled CyTOF and mIHC analyses. This research unraveled the dynamic immune landscapes related to the efficacy of ICIs in PLC patients, and constructed a prospective model that excelled in early discrimination of ICI‐responders.

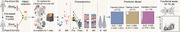

## 1 Introduction

Primary liver cancer (PLC), mainly encompassing hepatocellular carcinoma (HCC) and intrahepatic cholangiocarcinoma (ICC), represents a prevalent global malignancy and ranks as the third leading cause of cancer‐related mortality worldwide.^[^ ^1^ ^]^ HCC accounts for ≈90% of all primary liver cancer cases and commonly arises in the context of underlying chronic liver diseases such as alcohol‐related liver disease, chronic viral hepatitis (B or C), or non‐alcoholic fatty liver disease (NAFLD).^[^ ^1^ ^]^ Due to its insidious onset, the majority of patients present with advanced‐stage and unresectable PLC, leading to systemic therapy administration in more than half of the patients, including those with potentially resectable or advanced PLC.^[^ ^2^ ^]^ Presently, immunotherapy targeting the programmed cell death protein 1 (PD‐1)/programmed cell death ligand 1 (PD‐L1) axis, combined with anti‐vascular endothelial growth factor neutralizing antibody (anti‐VEGF) or tyrosine kinase inhibitors (TKIs), has emerged as a first‐ or second‐line therapy for advanced HCC patients, respectively.^[^ ^3^, ^4^ ^]^ As for ICC, PD‐1/PD‐L1 inhibitor‐based combine therapy has been proved by FDA as a first‐line systemic treatment in patients with advanced biliary tract cancer.^[^ ^5^ ^]^ Nevertheless, the therapeutic benefits remain limited, with objective response rates below 40%.^[^ ^6^ ^]^ A significant challenge that persists is the identification of predictive markers and treatment strategies for patients who do not respond to the current combinatorial immunotherapies.

Nowadays, there exists rapid progress in identifying the predictive biomarkers of ICI response, both to gain deeper insights into the mechanisms of resistance and to leverage the precision medicine approaches in immunotherapy for cancer. The commonly recognized biomarkers for immunotherapy response include the tumor mutation burden (TMB), microsatellite instability (MSI), PD‐L1 status (for certain cancer types), and certain mutated genes, such as mutant BRAF, KRAS and LKB1.^[^ ^7^ ^]^ The neutrophil‐to‐lymphocyte ratio (NLR), regulatory T cell‐to‐effector T cell ratio (Treg/Teff), and expression of oncofetal genes in situ have shown correlations with clinical outcomes in HCC patients undergoing Atezolizumab (anti‐PD‐L1) and bevacizumab (anti‐VEGF) combination therapy.^[^ ^4^ ^]^ Recent reports have also demonstrated the utility of single‐cell analysis in evaluating peripheral blood biomarkers for ICI response. Peripheral CXCR3^+^CD8^+^ TEM cells and APCs are reported to be independent predictors of response and PFS in patients with HCC treated with anti‐PD‐1 ICB.^[^ ^8^ ^]^ Other non‐invasive biomarkers, such as levels of IL‐6 and TGF‐*β*,^[^ ^9^ ^]^ AFP and CRP,^[^ ^10^ ^]^ were reported relevant to the prognosis of HCC patients treated with ICIs. However, the hindrance of clinical application for these TME biomarkers, and lack of ideal non‐invasive markers for predicting response, resulting in a scarcity of predictive biomarkers for ICIs therapy in PLC.

In our study, we employed cytometry by time of flight (CyTOF) to analyze immune cells in sequential PBMC samples and multiplex fluorescent immunohistochemistry (mIHC) on matched tumor and non‐tumor adjacent tissues from liver cancer patients treated with ICI‐based systemic therapy. We identified potential circulating candidate biomarkers for predicting response and resistance to ICI‐based therapy in PLC and described their characteristics within the tumor in situ. Additionally, we constructed a non‐invasively predictive model consisting of five parameters for assessing the efficacy of ICI‐based therapy.

## 2 Results

### 2.1 Patient Characteristics, Clinical Information Acquisition, and CyTOF Analyses Profile Comprehensive Immunological Landscape Before and During ICI‐Based Treatment

**Figure 1:**
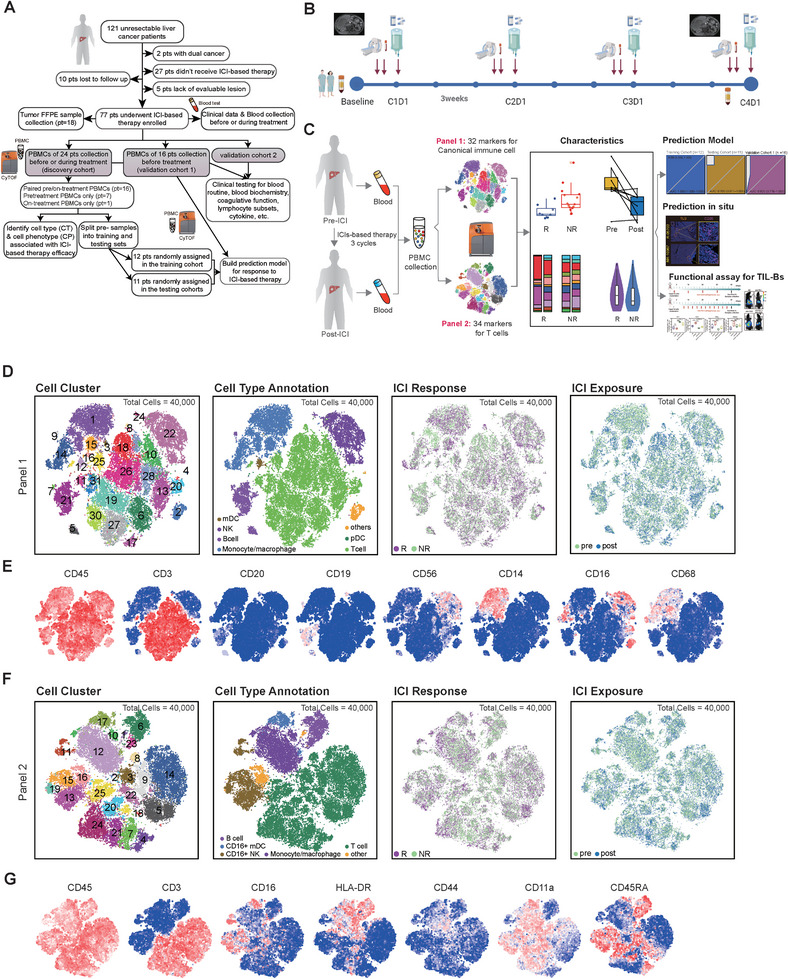
Patient characteristics, clinical information acquisition, and CyTOF analyses of comprehensive immunological features before and during ICI‐based treatment. A) Overview of subject enrollment, experimental design, clinical datasets, and analyses conducted in this study. Pts, abbreviation for patients. B) Schematic diagram illustrating the collection of samples. Immunotherapy using ICI‐based therapy is administered according to the indicated schedule (arrow). Time points for blood samples and radiographic data collection, as well as the CyTOF assays, are highlighted. C1D1, C2D1, C3D1, C4D1: First day of the initial, second, third and fourth treatment cycle, respectively. This panel was created using BioRender with an academic license. C) Graphical abstract presenting the workflow employed in its entirety. D) t‐SNE plots of Panel 1 displayed under different parameter settings. E) Representative examples of t‐SNE plots illustrating the normalized marker expression from all samples in Panel 1. F) t‐SNE plots of Panel 2 displayed under different parameter settings. G) Representative examples of t‐SNE plots depicting the normalized marker expression from all samples in Panel 2.

To interrogate whether the immune microenvironment of PBMCs can predict the response of PLC patients to ICI‐based therapy, a discovery cohort of 24 PLC patients was utilized, and a high‐throughput single‐cell‐level analysis workflow based on CyTOF was developed. The initial CyTOF analysis included a total of 40 cryopreserved PBMC samples obtained from 22 HCC and 2 ICC patients. These samples were collected at two‐time points: within 1 week before the initiation of treatment (referred to as “pre‐”) and 12 weeks after the start of treatment (referred to as “post‐”). Out of the enrolled patients, 23 provided pre‐treatment PBMC samples, and 17 provided post‐treatment PBMC samples. The clinicopathological features of the patients can be found in Tables S1 and S2 (Supporting Information). Responders were defined based on RECIST 1.1 criteria (Supplementary Materials and methods, Figure S1A, Supporting Information). Patients who showed better tumor regression had significantly longer overall survival (OS) and progression‐free survival (PFS) compared to non‐responders (**Figure** **1**A–C; Figure S1B, Supporting Information). Two separated but partially overlapping mass cytometry panels were designed (Figure 1C; Table S3, Supporting Information): One contained 32 markers to identify all of the canonical immune cell populations (panel 1, Figure 1D,E), and the other contained 34 markers that dedicated to the detailed profiling of T cell differentiation and activation (panel 2, Figure 1F,G).

After gathering a random sample of 1000 cells from each PBMC sample and a total of 40 000 cells from the CD45^+^ cell population, we applied dimensionality reduction techniques to visualize the high‐dimensional data in two‐dimensional t‐SNE plots. For panel 1, t‐SNE plots with 31 annotated immune clusters were obtained, while panel 2 resulted in t‐SNE plots with 25 annotated immune clusters (Figure 1D–G; Figure S1C,D and Table S4, Supporting Information).

Both the expression of functional molecules and the percentage of immune lineages (Figure S1F, Supporting Information) in each sample showed significantly positive correlations between the two panels (B cells: R = 0.95, p‐value <1×10^−4^; Monocytes: R = 0.98, p‐value <1×10^−4^; T cells: R = 0.97, p‐value <1×10^−4^), demonstrating high‐test stability of the two individual designed panels and our workflow. Likewise, given that two independent batches were prepared and run separately, the homogeneity of each marker's expression between the two experiments in all major immune subsets were also queried, and the results demonstrated that there was barely any batch effect in the present data (Figure S1G, Supporting Information).

### 2.2 Baseline Canonical Immune Features and their On‐Therapy Dynamics in PBMCs Reflect the Clinical Response to ICI‐Based Therapy

**Figure 2:**
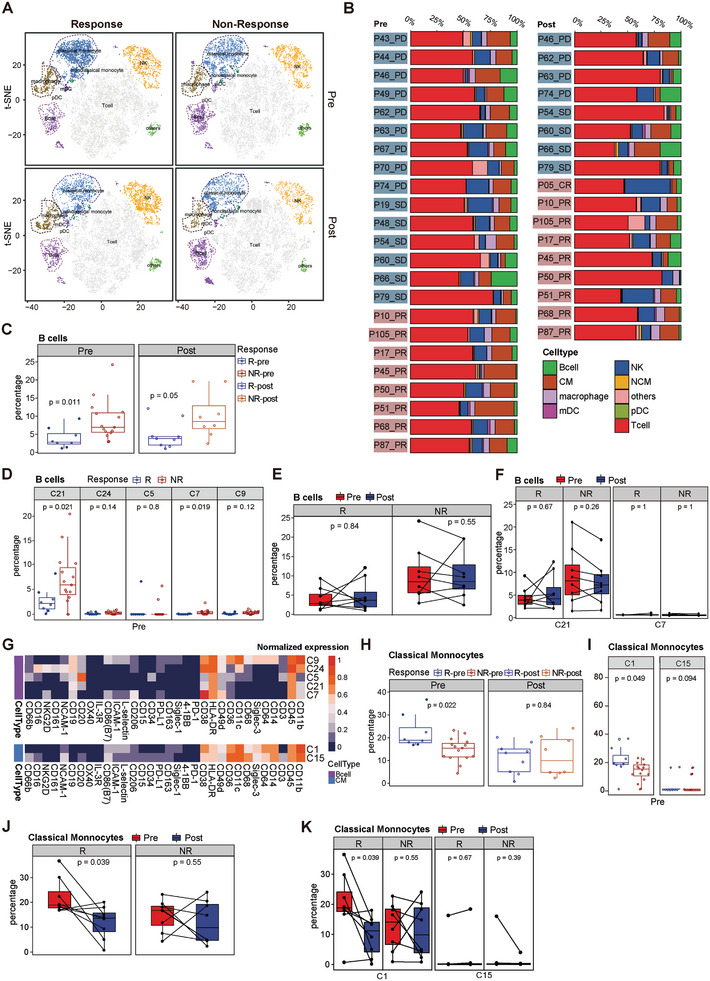
Baseline canonical immune features and their on‐therapy dynamics in PBMCs reflect the clinical response to ICI‐based therapy. A) t‐SNE plots of panel 1 (23 pre‐treatment samples and 17 post‐treatment samples) showed that 9 classic immune cell types were identified in total PBMCs via phenograph clustering method. B) Frequencies of classic immune subsets are depicted for each sample pre‐ and post‐treatment. The analysis comprised samples of 8 responders (Rs) and 15 non‐responders (NRs) in the pre‐treatment cohort and 9 Rs and 8 NRs in the post‐treatment cohort, respectively. C) Cell percentages of B cells are plotted and compared between Rs and NRs both pre‐treatment (left) and post‐treatment (right). D) Cell percentages of subclusters of B cells (C21, C24, C5, C7, and C9) are plotted and compared between Rs and NRs pre‐treatment. E,F) Dynamic changes in the percentage of B cells (E) and its subclusters (F) before (pre) and after (post) ICI‐based therapy are depicted in patient‐matched PBMCs from 8 Rs and 8 NRs. G) Heatmap displaying the mean expression level of all 32 markers in 7 subclusters of B cells and classical monocytes (CMs), respectively. H) Cell percentages of CMs are plotted and compared between Rs and NRs, pre‐and post‐treatment. I) Cell percentages of subclusters of CMs (C1 and C15) are plotted and compared between Rs and NRs pre‐treatment. J,K) Dynamic changes in the percentage of CMs (J) and its subclusters (K) before (pre) and after (post) ICI‐based therapy are depicted in patient‐matched PBMCs from 8 Rs and 8 NRs. The line and box represent the mean and upper and lower quartiles, respectively. Wilcoxon rank‐sum test and paired Wilcoxon signed rank test (for paired data) were used to identify significant differences.

Utilizing the 31 clusters identified in panel 1, we further classified the classic immune subsets, including T cells, B cells, CD14^+^CD16^+^ non‐classical monocytes (NCMs) or CD14^+^CD16^−^ classical monocytes (CMs), macrophages, natural killer (NK) cells, plasmacytoid dendritic cells (pDCs), myeloid dendritic cells (mDCs), and other lineages (**Figure** **2**A; Figure S2A,B and Table S4, Supporting Information). Cell frequencies were calculated for each sample, and the composition of individual samples was represented (Figure 2B). Furthermore, the normalized molecular expression of each cluster was depicted (Figure 2A,B; Figure S2A and Table S5, Supporting Information).

The analysis of the classic immune lineages revealed a significant variation in their composition among patients (Figure 2B), indicating a high level of heterogeneity in the interpatient immune microenvironment within PBMCs. Noteworthily, the number of B cells (Figure 2C) showed a progressive reduction in responders, whereas CD14^+^CD16^−^ classical monocytes exhibited the opposite trend (Figure 2H). Using CyTOF, five distinct clusters of B cells (C5, C7, C9, C21, and C24) were identified (Figure 2D). Among them, the proportion of cluster 21 (C21) in baseline PBMCs of responders was 38% of non‐responders with a statistically significant difference, and C21 was the predominant subcluster of peripheral B cells (≈74.3%) (Figure 2D; Figure S2C, see Figure S1A, Supporting Information for gating). This tendency was sustained at 12 weeks after ICI‐based therapy (Figure 2E,F), that is, the quantity and composition of the B cells remained similar after the ICI‐based therapy. Cluster 21 exhibited deficient expression of CD33, CD161, CD38, CD206, CD86, ICAM‐1, and intermediate expression of HLA‐DR, indicating a population of naïve and incompetent B cells (Figure 2G and data not shown).

In contrast, analysis of CD14^+^CD16^−^ classical monocytes, primarily Cluster 1 (CM_C1, CD68^low^NCAM^−^ classical monocytes, Figure 2G), revealed a 1.5‐fold enrichment in the baseline PBMCs of the responder group compared to the non‐responder group (Figure 2H,I; Figure S2C, Supporting Information). Specifically, the proportion of monocytes (mainly C1) in responders PBMCs exhibited a sharper decline after exposure to ICI‐based therapy (Paired Wilcoxon signed rank test, p‐value = 0.039 for both CM cells and cluster 21, Figure 2J,K), suggesting that the number of classical monocytes could be influenced by the therapy, with a greater decrease post‐treatment associated with a better response. No intergroup differences were observed in the remaining immune clusters or PBMC subpopulations at baseline. Though when considering changes in immune composition during treatment, the proportion of macrophages (C14) showed a significant reduction of ≈10% in ICI‐resistant individuals following therapy (Figure S2D, Supporting Information). Other than that, the ratio of mDCs in PBMCs was significantly diminished after ICI‐based therapy, with the change being more pronounced in responders compared to non‐responders (Figure S2E, Supporting Information, p = 0.04 in the whole cohort, p = 0.05 in the responder group). These findings support the concept that ICI therapy induces varying shifts in different immune populations within PBMCs of PLC patients. Moreover, the baseline ratios of B cells and classical monocytes, as well as the dynamic changes in classical monocytes during the early stage of ICI‐based therapy, may serve as potential predictors of responsiveness in PLC patients.

### 2.3 The Majority of Immune Lineages in the ICI‐Responsive Group Exhibit Simultaneous High Expression Levels of Cell Adhesion, Activation, and Exhaustion Molecules

**Figure 3:**
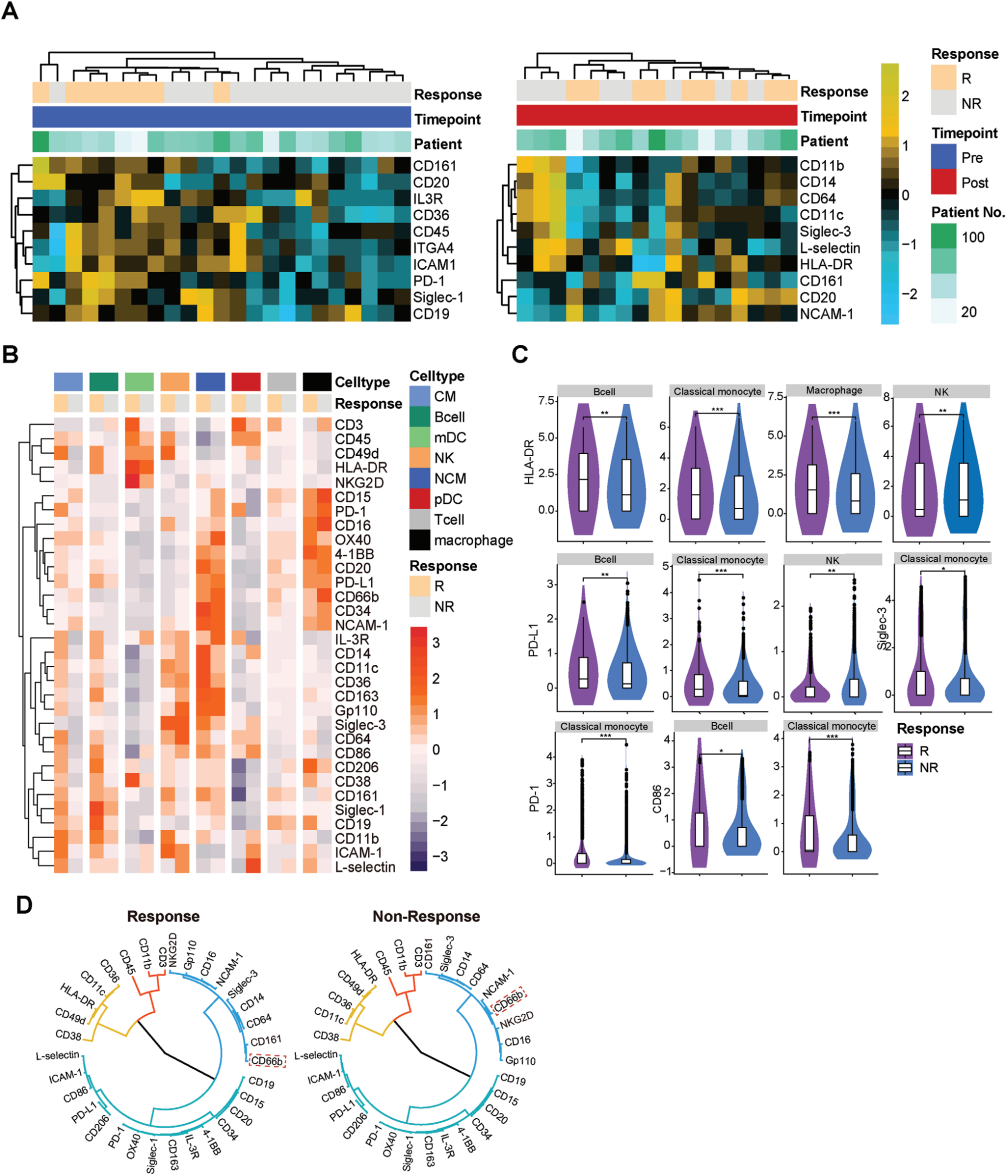
The majority of immune lineages in the ICI‐responsive group exhibit simultaneous high expression levels of cell adhesion, activation, and exhaustion molecules on their surface. A) Heatmap displaying the scaled normalized expression for 10 markers that showed the greatest differential expression between responders (Rs) and non‐responders (NRs) before (left panel) and 12 weeks after (right panel) initiation of therapy. Median expression was calculated on single, live CD45^+^ cells derived from thawed PBMC samples obtained from patients. Dendrograms for markers (rows) and samples (columns) were generated using hierarchical clustering with Euclidean distance. Bars at the top of the heatmap represent individual samples from Rs (orange) and NRs (grey). Each column represents a patient sample from a specific time point, with a total of 23 patients before treatment and 17 patients after treatment. B) Heatmaps illustrating the normalized median expression of the 32 markers from panel 1 in each patient's indicated immune cell subtypes before treatment. Expression was quantified using Salmon and represented as a fold change relative to the median value of gene expression for each patient. C) Violin plots illustrating the normalized median expression of the differentially expressed markers from panel 1 in each cell of the indicated immune cell subtypes. A comparison of the expression levels of several surface markers (HLA‐DR, PD‐L1, PD‐1, Siglec‐3) between the indicated cell types from the pre‐treatment R and NR groups was performed using a two‐sided Wilcoxon rank‐sum test. *p < 0.05, **p < 0.01, ***p < 0.001. D) Clustering tree for the relationships among all 32 surface markers in Rs and NRs before ICI‐based therapy.

We performed hierarchical clustering using the top‐ten markers with the distinguishing expression on peripheral CD45^+^ cells from each patient (Table S6, Supporting Information). Our analysis showed two major clades of baseline samples: one branch (right) consisted of 10 non‐responder patients, while the other branch (left) included 13 patients, of whom 8 (62%) were responders (**Figure** **3**A, left panel). Nevertheless, hierarchical clustering of exposed PBMCs failed to differentiate responders from non‐responders (Figure 3A, right panel). This prompted us to execute a more in‐depth analysis.

Analysis for molecular expression profile showed higher expression of nearly all cell adhesion molecules (CAMs) (Siglec‐1, IL‐3R, PD‐1, CD14, CD86, PD‐L1, ICAM‐1, CD64, CD34, and CD206) on classical monocytes of responders compared to non‐responders, indicating a tumor‐infiltrating phenotype of these cells. Remarkably, circulating B cells in beneficiaries exhibited increased expression of immunosuppression/exhaustion markers PD‐1 and PD‐L1, possibly indicating the vulnerability to PLC in ICI‐based therapy (Figure 3B).

We additionally performed a differential expression analysis for surface markers on each CD45^+^ cell (data in Table S4, Supporting Information). Consistent with published data showing better response to anti‐PD‐1 therapy in tumors with PD‐1/PD‐L1^high^ TILs,^[^ ^11^ ^]^ our gene expression analysis revealed elevated levels of PD‐L1 and PD‐1 in B cells and classical monocytes of patients benefiting from ICI‐based therapies (Figure 3C). HLA‐DR, a marker of antigen presentation, showed higher expression on B cells, classical monocytes, and macrophages in responders compared to non‐responders (Figure 3C). Additionally, cell adhesion and activation markers such as CD38, CD64, Siglec‐1, and CD206 (Figure S3, Supporting Information), were predominantly upregulated on immune subpopulations in the ICI‐respondent cohort. The co‐expression pattern of cell surface markers in the R and NR groups is summarized in Figure 3D. Before ICI administration, the expression of CD66b (neutrophil marker) on CD45^+^ PBMCs correlated closely with CD161 in the R group, while in the NR group, CD66b co‐expressed with NCAM‐1, indicating distinct neutrophil features may have differential impacts on immunotherapy efficacy.

Taken together, the immune‐related gene signatures from our study highlighted that patient who respond to ICI‐based therapy exhibited a peripheral microenvironment enriched with activated immune cells, which simultaneously expressed immune checkpoint markers as well as molecules of cell adhesion, antigen presentation, and cell co‐stimulation.

### 2.4 T Cell Clustering and Subtype Analysis Reveal the Diversities of HLA‐DR^+^CD8^+^ T Subcluster Between the Responders and Non‐Responders

**Figure 4:**
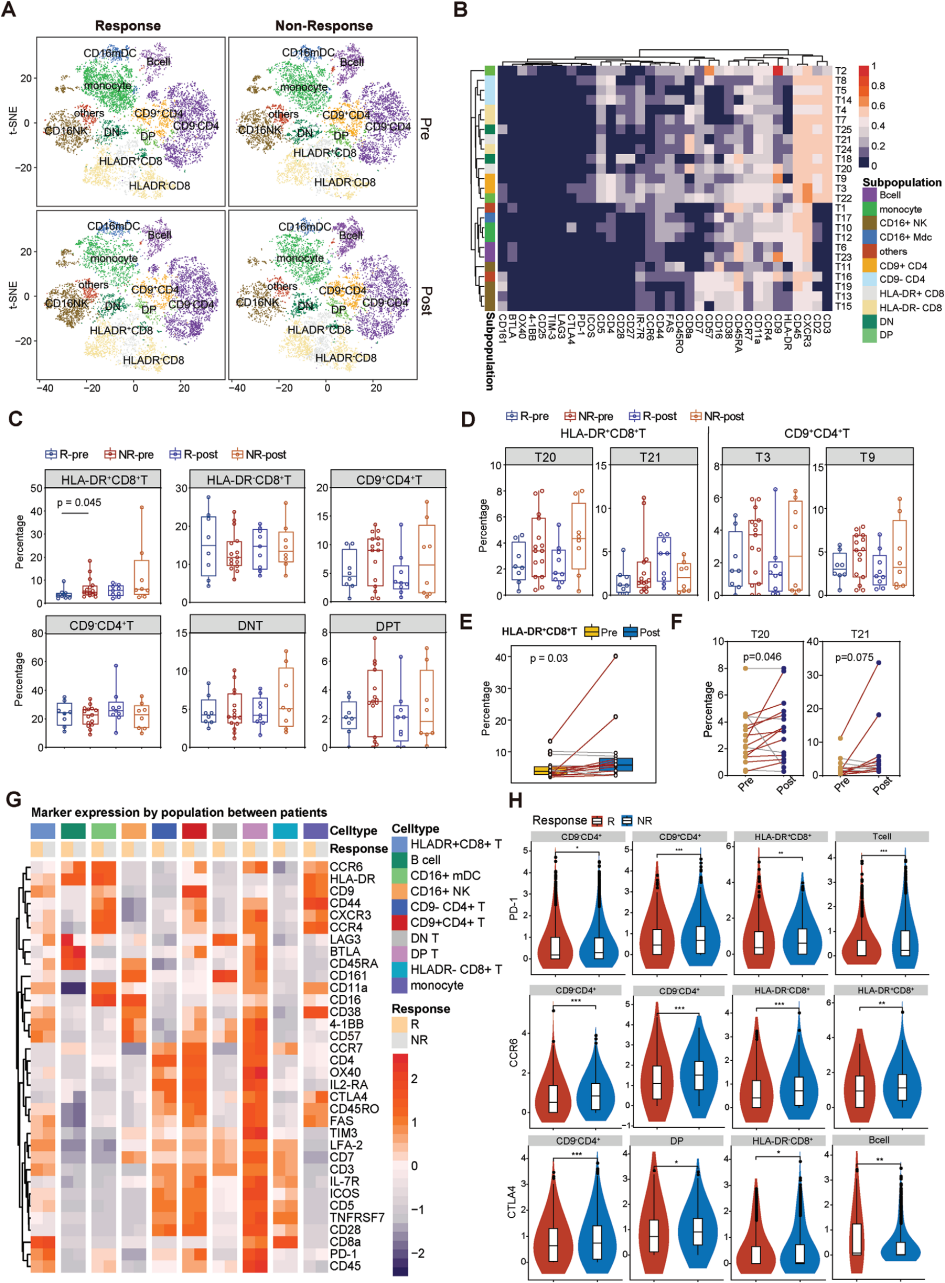
T cell clustering and subtype analysis reveal the diversities of HLA‐DR^+^CD8^+^ T subcluster between the responders and non‐responders. A) t‐SNE plots of panel 2 (23 pre‐treatment samples and 17 post‐treatment samples) show the identification of 6 T cell subpopulations and 5 non‐T cell subpopulations in total PBMCs using the phenograph clustering method. B) Heatmap displaying the mean expression level of all 34 markers in 25 clusters and 11 cell subpopulations. C) Cell percentages of the indicated cell subpopulations are plotted and compared between Rs and NRs both pre‐treatment and post‐treatment. D) Cell percentages of the subclusters of HLA‐DR^−^CD8^+^ T cells (T20 and T21) and CD9^+^CD4^+^T cells (T3 and T9) are plotted and compared between Rs and NRs both pre‐treatment and post‐treatment. The line and box represent the mean and upper and lower quartiles, respectively. Wilcoxon rank‐sum test was used to identify significant differences. No significant differences were observed. E,F) Dynamic changes in the percentage of HLA‐DR^−^CD8^+^ T cells (E) and their subclusters (F) before (pre) and after (post) ICI‐based therapy are depicted in patient‐matched PBMCs from all 16 PLC patients. Paired Wilcoxon signed rank test was used to identify significant differences. G) Heatmaps illustrating the normalized median expression of the 34 markers from panel 2 in each patient's indicated immune cell subtypes before treatment. Expression values were quantified using Salmon and represented as a fold change relative to the median value of gene expression for each patient. H) Violin plots illustrating the normalized median expression of the differentially expressed markers from panel 2 in each cell of the indicated immune cell subtypes. A comparison of the expression levels of several surface markers (CTLA‐4, PD‐1, CCR6, BTLA) between the indicated cell types from the pre‐treatment R and NR groups was performed using a two‐sided Wilcoxon rank‐sum test. *p < 0.05, **p < 0.01, ***p < 0.001.

To comprehensively characterize T cells, we designed a specialized CyTOF panel with 34 markers (Panel 2, Table S3, Supporting Information) and employed FlowSOM to identify 25 distinct immune subclusters (T1–T25) (Figure S4A,B, Supporting Information). Given that changes in traditional T cell subpopulations did not differ between responders and non‐responders (Figure S4A,B, Supporting Information, and data not shown), we reclassified CD4^+^ T cells based on their cell surface molecular expression characteristics as CD9^+^CD4^+^ and CD9^−^CD4^+^T cells. Additionally, we categorized CD8^+^ T cells based on the activation marker HLA‐DR (**Figure** **4**A,B; Figure S4A‐D and Table S7, Supporting Information). Similarly, CD3^+^ T cells, including CD8^+^T, CD4^+^T, CD4^+^CD8^+^ dual‐positive T cells (DPT), and CD4^−^CD8^−^ dual‐negative T cells (DNT), constituted the major population in PBMCs (Figure S4E, Supporting Information). In parallel, for the annotated cell subpopulations, cell frequencies were computed in each sample, the composition of the individual samples and the molecular expression of each cluster were also depicted (Figure 4A,B; Figure S4C‐E and Table S8, Supporting Information).

Then we interrogated the most prominently differentiated clusters of T cells between the R and NR groups, before and after ICI‐based treatment. At baseline, merely the HLA‐DR^+^CD8^+^ T cells showed a 1.5‐fold significant increase in the non‐responders (p = 0.045, Figure 4C), whereas the distribution of all other T cell subpopulation had no statistical difference between the two groups, regardless of timepoint (Figure 4C,D). Intriguingly, all HLA‐DR^+^ CD8^+^ T clusters concomitantly highly expressed CD38, and none of the HLA‐DR‐negative clusters expressed CD38 (Figure 4B). Given that the NAD enzyme CD38 has received much attention as a biomarker of CD8^+^ T‐cell exhaustion,^[^ ^12^, ^13^ ^]^ we presumed that CD38 plays an essential role for the peripheral blood microenvironment of immunotherapy‐resistant patients for the HLA‐DR^+^CD8 T cell subpopulation. Notably, the number of peripheral HLA‐DR^+^CD8^+^ T cells (p = 0.029, Figure 4E), especially the CD57^+^HLA‐DR^+^CD8^+^T (T20, p = 0.046, Figure 4F; Figure S4F, Supporting Information), exhibited a uniform increase in 12 out of 16 patients after 9 weeks of ICI‐based therapy, irrespective of treatment responding. T20 represents an HLA‐DR^+^CD8^+^T subcluster characterized by moderate PD‐1 along with high CD45RA and CD57 expression (Figure 4B), akin to “exhausted senescent” TEMRA (Effector Memory‐Expressing CD45RA) T cells.^[^ ^14^ ^]^

To elucidate the potential role of T cells more thoroughly, we separately characterized and annotated 18 T cell clusters (TC1–TC18) in panel 2 based on the 34 markers (Figure S5A, Supporting Information, only show the annotation results of T cells in panel 2). As shown in Figure S5B (Supporting Information), the proportion of circulating TC10, which defined as CD45^+^CD57^−^CD8^+^ T cells that were naïve and could predict a worse PFS in pancreas cancer patients,^[^ ^15^ ^]^ was higher at baseline in NR patients than in R patients of PLC. What's more, the proportion of circulating CD8^+^Tcm (TC4, Figure S5A, Supporting Information) significantly declined after treatment in immunotherapy‐refractory patients (Figure S5D, Supporting Information). Altogether, these results denoted a higher prevalence of naïve, as well as the senescent CD8^+^ T subpopulations in the PBMCs of non‐responders prior to treatment, and the number of these specific T subclusters rose after the implementation of ICI‐based therapy. Such phenomenon suggested that the induction or maintenance of CD8^+^ memory T cells and inhibition of CD8^+^ naïve or exhausted T cells could be a promising treatment strategy to ensure the efficacy of immunotherapy.

### 2.5 Immuno‐Suppressive Phenotypes of HLA‐DR^+^CD8^+^ T Cells are Related to the Resistance to ICI‐Based Therapy

Next the relationship between the expression of functional markers on T cells and their response to ICI‐based therapy was investigated. Regrettably, the hierarchical clustering approach based on the top‐10 differentially expressed surface markers in the T cell repertoire (Figure S6A, Supporting Information) did not enable effective stratification of ICI‐naïve and ICI‐exposed patients into responder and non‐responder groups. Moreover, the use of simple cell lineage classification alone was still insufficient to differentiate between responders and non‐responders (Figure S6B,C, Supporting Information).

At the patient level, it is observed that the fold changes of the inhibitory receptor PD1 and CD161 expression were 1.5 and 1.4 in HLA‐DR^+^CD8^+^T cells of the non‐responders than responders, respectively. On the contrary, levels of both cytotoxic marker CD16^[^ ^16^ ^]^ and co‐stimulation marker CD27 on these T cells receded over 20% in this CD8^+^ subpopulation from non‐responders (Figure 4G; Table S9, Supporting Information). These findings further highlight the presence of an inactivated and exhausted T cell phenotype in non‐responders.

At the single‐cell level in non‐responders, nearly all immuno‐suppressive/exhausted markers as PD‐1, CTLA4, and BTLA, along with CCR6, a receptor comprising CCR6/CCL20 axis and promoting cancer progression,^[^ ^17^ ^]^ elevated consistently in peripheral HLA‐DR^+^CD8^+^ T cells (Figure 4H; Figure S6D and Table S7, Supporting Information). The co‐expression pattern of the T cell surface markers in the R and NR groups before treatment are presented in Figure S6E (Supporting Information). In the responders, the expression of CTLA‐4 was more closely related to co‐stimulating and proliferating markers, such as CD27, ICOS, and HLA‐DR, suggesting an immune activation state of the T cells. However, in the non‐responders, CTLA4 co‐expressed with bunches of other immune‐checkpoints (LAG3, PD1, BTLA, and TIM3), which implied that the state of the T cells was more suppressed than those of the responders.

In conclusion, our analysis revealed the presence of a functionally impaired HLA‐DR^+^CD8^+^T subpopulation in the peripheral circulation of non‐responders to ICI‐based therapy. These findings have significant implications as potential biomarkers for predicting therapeutic efficacy and identifying vulnerable targets for immunotherapy in patients with primary liver cancer.

### 2.6 Correlation Across Immune Profile and Clinical Characteristics of ICI‐Treated PLC Patients

**Figure 5:**
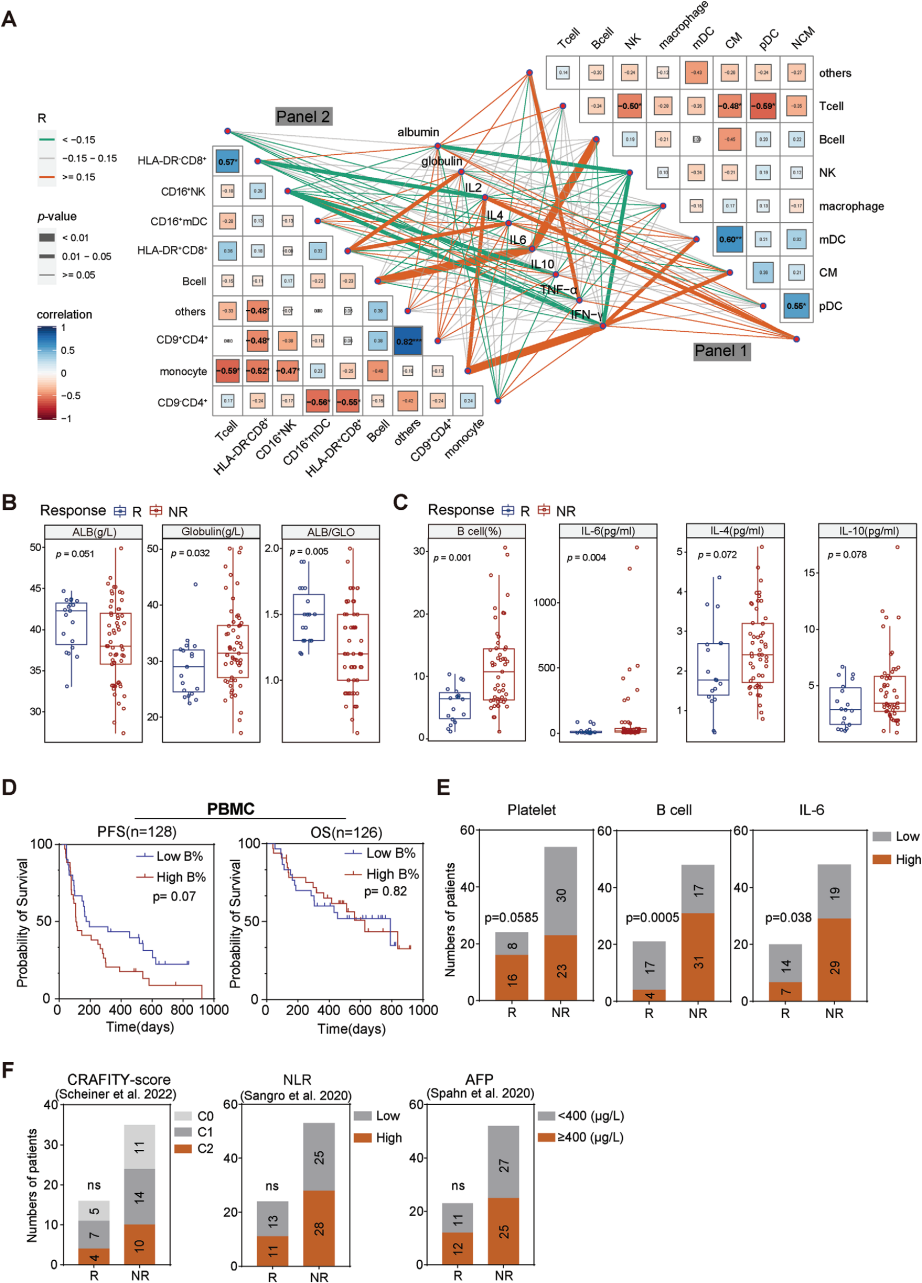
Correlation between immune profile and clinical characteristics of ICI‐treated PLC patients. A) Pearson correlation analyses depicting the relationship between serum levels of clinical biomarkers and the frequencies of cell subpopulations from panel 1 (right) and panel 2 (left) in the discovery cohort. Distance measurement was performed using Euclidean distance. Statistical significance is indicated by asterisks (*p < 0.05, **p < 0.01, ***p < 0.001). B,C) Comparison of serum levels of clinical biomarkers between Rs and NRs pre‐treatment. B, the analysis included a total of 76 patients for whom blood routine and blood biochemistry were tested in the clinical laboratory. C, the analysis included a total of 68 patients for whom the classical immune lineages from PBMC samples were tested in the clinical laboratory. D) Kaplan–Meier survival analysis for OS and PFS of PLC patients with high or low B cells, median B‐cell percentage was defined as the cutoff value. The analysis comprised 64 patients who were included based on the availability of B‐cell percentage data from laboratory measurements and follow‐up survival data. E) Association between circulating platelet counts (left), proportion of B cells (middle), level of IL‐6 (right), and therapeutic response, respectively. Two‐tailed chi‐square test was used to determine the statistical significance between the groups. F) Association between circulating CRAFITY‐score (left), NLR (middle), levels of AFP (right), and therapeutic response, respectively. Sample size for each analysis was labelled on the histogram. ns, not significantly.

To further explore the clinical implications of the immune profile in the peripheral blood before treatment, we queried the clinical relevance of specific immune spectrums in peripheral blood. In the cohort that underwent CyTOF testing, we observed a positive correlation between the fraction of classical monocytes and mDC (Pearson correlation coefficient was 0.6, p < 0.01), while a negative correlation was observed between monocytes and several other subpopulations, for instance, CD16^+^NK cells and T cells (especially the HLA‐DR^−^CD8^+^ counterpart) (**Figure** **5**A). Accordantly, the proportions of B cells from both panels were positively correlated with the level of IL‐6 in baseline plasma. We also found that IFN‐*γ*, a cytokine known to promote antigen presentation and activates immune cells,^[^ ^18^ ^]^ was positively associated with the classical monocyte of panel 1 and monocyte from panel 2, respectively. Moreover, we identified an inverse correlation between the proportion of NK cells and plasma albumin (ALB) levels, as well as a positive association between HLA‐DR^+^CD8^+^ T percentages and globulin levels (Figure 5A).

In pursuit of clinical features associated with the effectiveness of ICI‐based therapy in liver cancer, an up‐scaled cohort consisting of 77 ICI‐treated patients was established (Tables S1, S2, and S10, Supporting Information). Clinical parameters for each patient were collected before and during the ICI treatment process. Congruently, patients with better performance on immunotherapy had lower globulin levels, higher albumin levels and albumin/globulin ratio (Figure 5B). This was legitimate, as the patients with advanced PLC are often suffering from liver cirrhosis and dysfunction, frequently manifesting as hypoalbuminemia.

In line with the findings from CyTOF analysis, the clinical laboratory data revealed a higher percentage of B cells in the PBMCs of non‐responders before treatment, further supporting the notion that peripheral B cells may disrupt ICI‐based therapy (Figure 5C). Although the abundance of B cells did not show a significant association with OS, patients with a lower percentage of B cells possessed a non‐significant yet prolonged median PFS (170 days vs 96 days, p = 0.07, Figure 5D). IL‐6, a cytokine whose blockade was discovered to abrogate immunotherapy toxicity and promote tumor immunity,^[^ ^19^ ^]^ was unsurprisingly higher in the plasma of non‐responders (Figure 5C). Reciprocally, serum IL‐4, IL2, and IL‐10 were also showed increasing tendency whilst INF‐*γ* decreased in the NR group, despite the insignificance (Figure 5C; Figure S7A, Supporting Information).

Patients with more B cells and higher IL‐6 levels in blood had significantly lower ORR than those with less peripheral B cells and IL‐6 (B cell: 11.4% vs 50.0%, p = 0.0005; IL‐6: 19.4% vs 42.4%, p = 0.038) (Figure 5E). As previous studies suggested that neutrophil‐to‐lymphocyte ratio (NLR),^[^ ^4^ ^]^ C‐reactive protein (CRP) and AFP in immunotherapy (CRAFITY)^[^ ^10^ ^]^ may predict response of immunotherapy, we explored the correlation between these parameters and efficacy. There was neither correlation between NLR, AFP level, CRAFITY score and ORR (NLR: 28.2% vs 34.2%, p = 0.32; AFP: 32.4% vs 28.9%, p = 0.74; CRAFITY Score: 28.6% vs 33.3% vs 31.25%, p = 0.09), nor between tumoral PD‐L1 status and ORR (PD‐L1: 40% vs 37.5%, p = 0.91) in our cohort (Figure 5F; Figure S7B, Supporting Information).

**Figure 6:**
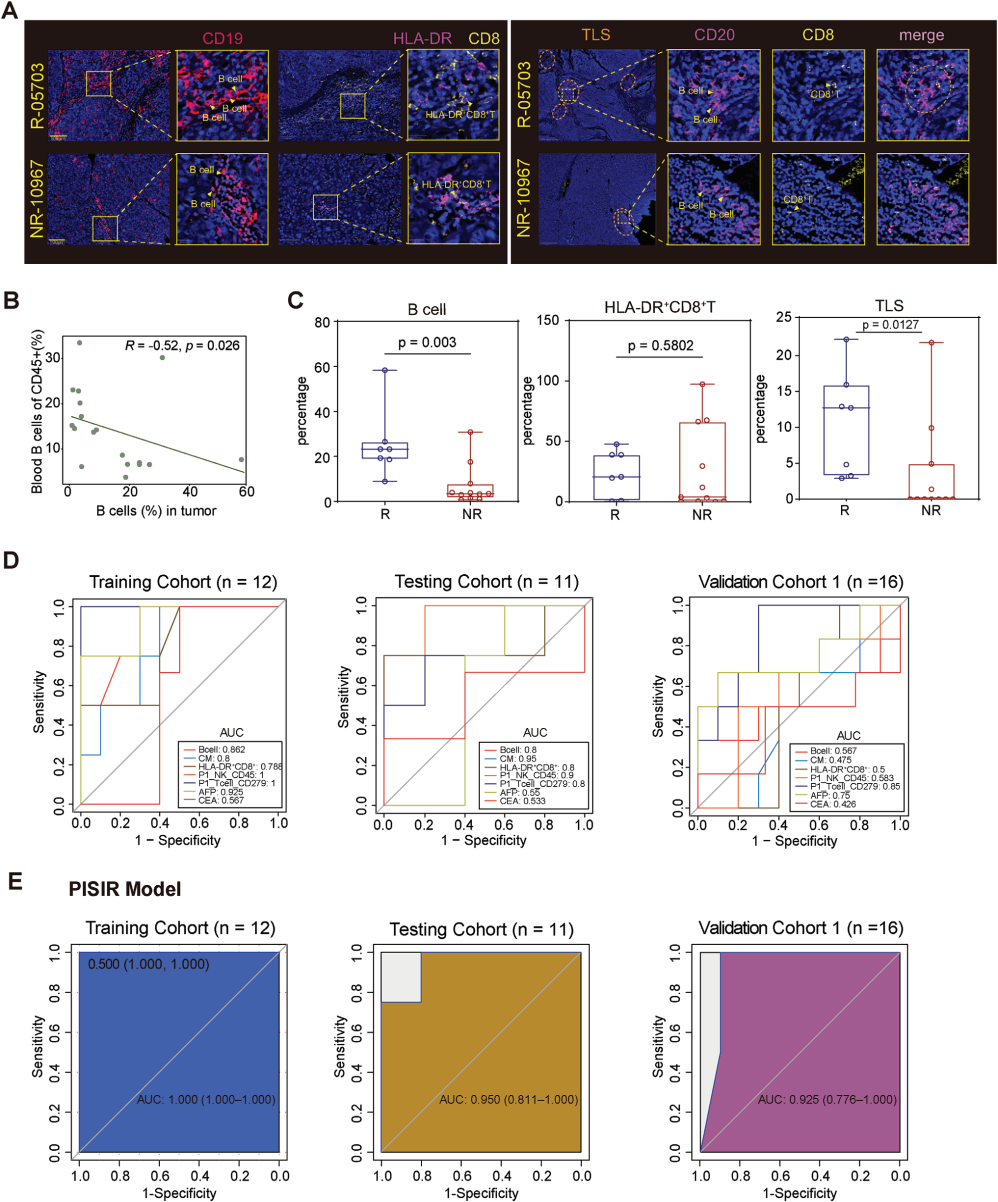
Prediction model PISIR for identifying promising responses in PLC patients based on the peripheral immune signature of PBMCs before ICI‐based therapy. Representative image of mIHC detection in PLC samples, illustrating HLA‐DR^+^CD8^+^ T cells, B cells (left panel) and tumor‐infiltrating lymphoid structures (TLS, right panel) from 18 patients with different responses to ICI‐based therapy in the corresponding FFPE tumor samples. B) Pearson correlation analyses between the blood and tumor in situ frequencies of B cells in the 18 patients from mIHC analysis. C) Comparison of the percentages of B cells and HLA‐DR^+^CD8^+^ T cells in CD45^+^ cells in situ, as well as the counts of TLS per square centimeter, between Rs (n = 7) and NRs (n = 11) pre‐treatment. The line and box represent the mean and upper and lower quartiles, respectively. D) Comparison of multiple ROC plots showcasing the performance of single clinical parameters and immune profiles in the training cohort (left), testing cohort (middle), and validation cohort 1(right). E) ROC plot depicting the performance of the PISIR model in the training cohort (left), testing cohort (middle), and validation cohort 1 (right).

As tumor B lymphocyte infiltration is closely linked to the presence of intra‐tumoral tertiary lymphoid structures (TLS), we further executed multiplex fluorescent immunohistochemistry staining using 18 therapy‐naïve PLC tumor samples. The quantities and localization of CD19^+^B cells, HLA‐DR^+^CD8^+^T cells, and the presence of TLS (identified by co‐localization of CD20^+^B cells and CD8^+^T cells) were shown in **Figure** **6**A and Table S11 (Supporting Information). We discerned a significantly negative correlation between the percentage of B cells (R = −0.52, p = 0.026), but not HLA‐DR^+^CD8^+^T cells, in the peripheral circulation and within the tumor microenvironment (Figure 6B and data not shown). Compared to non‐responders, responders had a twofold higher enrichment of B cells or TLS within their tumors, whereas the number of HLA‐DR^+^CD8^+^T cells didn't show a notable difference between the two groups (Figure 6C). Survival analysis also showed that patients with a higher proportion of B cells in tumors possessed prolonged PFS and OS (Figure S7D, Supporting Information). Median OS was not reached in the low‐B% group and 412 days in the high‐B% group (Hazard Ratio [HR], 0.09; 95% CI of ratio, 0.012–0.687; log‐rank p = 0.02). Median PFS was 569 days in the low‐B% group and 142 days in the high‐B% group (HR, 0.28; 95% CI of ratio, 0.089–0.868; log‐rank p = 0.028). Patients with denser TLS had a survival benefit in terms of PFS and OS, but the difference was not statistically significant (Figure S7E, Supporting Information). Of particular interest, survival data from both our ICI‐therapy cohort and TCGA‐LIHC cohort demonstrated that enrichment of tumor‐in‐situ HLA‐DR^+^CD8^+^T cells portended a better prognosis for HCC patients (Figure S7F,G, Supporting Information).

To delineate the potential role of tumor‐infiltrating B (TIL‐B) cells more clearly in the anti‐PD‐1 treatment for PLC, we accomplished an orthotopic tumor transplantation mice model in the liver using Hepa1‐6 cells and treated the mice with anti‐CD20 antibody, anti‐PD‐1 antibody, and a combination of the two, respectively (Figure S8A, Supporting Information). Comparable to the results for other tumor types, administration of anti‐CD20 antibody to mice reduced the therapeutic efficacy of anti‐PD‐1antibody against liver tumor of Hepa1‐6 cells (Figure S8A‐C, Supporting Information), as well as inhibited proliferation (Ki‐67) and tumor‐killing activity by downregulating IFN‐*γ*, Perforin and upregulating PD‐1 of the tumor‐infiltrating CD8^+^T cells (Figure S8D‐F, Supporting Information). Besides, we further explored the function of TIL‐B cells using the in vitro co‐culture system. To our expectation, B cells sorted from Hepa1‐6 orthotopic transplanted liver tumors can augment the efficiency of tumor killing by T cells and strengthened the efficacy of anti‐PD‐1 in vitro (Figure S8G, Supporting Information), possibly also through increased proliferation, strengthened expression of IFN‐*γ* and Perforin, and down‐regulation of PD‐1 expression in CD8^+^T cells (Figure S8H, Supporting Information).

These findings collectively suggested that both circulating and TIL‐B cells, may play crucial immunomodulatory roles and directly impact the efficacy of immunotherapy in liver cancer, with potentially heterogeneous functions. The presence of intra‐tumoral B cells, TLS, and HLA‐DR^+^CD8^+^T cells may have important implications for understanding the tumor immune microenvironment and its interaction with immunotherapeutic interventions.

### 2.7 Prediction Model PISIR for Identifying Responses in PLC Patients Based on the Immune Signature of PBMCs Before ICI‐Based Therapy

To deeply investigate the predictive value of the immune landscape in ICI‐based therapy for PLC, we divided the patients previously analyzed by CyTOF into two distinct datasets randomly: a training cohort (n = 12) and a testing cohort (n = 11). The performance of predictive models was evaluated using receiver‐operating characteristic curve (AUC) values, considering several single immune parameters with inter‐group differences, as well as AFP and CEA, which are commonly used tumor markers in clinical practice to assess treatment efficacy. However, these models exhibited variable and non‐robust AUC values across the datasets, ranging from 0.426 to 1 (Figure 6D). To overcome this limitation, we utilized the most differentially enriched immune cell populations and expressed markers to establish a comprehensive response‐predicting model for PLC patients treated with ICIs, referred to as the “peripheral immune signature of ICI‐response” (PISIR). Noteworthily, the PISIR model demonstrated a remarkable AUC value of 1.0 in the training cohort (Figure 6E). The PISIR model was constructed using the following formula: Response =  0.57×CM% – 5.53×Bcell% – 3.62×HLA‐DR^+^CD8^+^T% + 70.81×NK (CD45exp) + 484×Tcell (CD279exp) – 367.24 (Figure 6E). The output of the PISIR model is a categorical variable, where a value of 0 represents ICI‐resistance and a value of 1 represents ICI responsiveness. To authenticate the performance, we collected extra samples from an independent validation cohort 1, and the PISIR model achieved a satisfactory AUC of 0.95 and 0.925 in the testing cohort and validation cohort 1, respectively (Figure 6E; Tables S12–S15, Supporting Information). These data formulated the potential utility of this combined model PISIR in predicting ICI‐related response in PLC, using only five peripheral parameters.

## 3 Discussion

Immune‐checkpoint inhibitors (ICIs), represented by PD‐1/PD‐L1 inhibitors, are deemed a milestone and have become an indispensable part of the standard regimen for advanced hepatobiliary tumors. Nevertheless, their effectiveness remains limited, with an objective response rate of only ≈30% even when combined with other regimens.^[^ ^6^ ^]^ Circulating biomarkers in peripheral blood, such as NLR^[^ ^4^ ^]^ and CRAFITY score^[^ ^10^ ^]^ were reported to be relevant to the clinical response of immunotherapy. Besides, tumor immune microenvironment (TiME) is also closely related to the efficacy of immunotherapy and could be a valuable potential biomarker of immunotherapy.^[^ ^4^ ^]^ Nonetheless, these efforts on biomarkers for immunotherapy have not yielded steady and easily translatable results.

In our study, using single‐cell CyTOF and mIHC analyses, we portrayed a comprehensive and dynamic immunological atlas associated with response and prognosis in advanced PLC patients who underwent ICI‐based regimens, most of which were combinational immunotherapies with widespread clinical use. Herein for the first time, we established a cohort with ICI‐treated advanced PLC patients and identified three baseline characterizations – higher CD14^+^CD16^−^ classical monocyte, lower B cell and HLA‐DR^+^CD8^+^ T cell abundance in the peripheral blood – as promising determinants of ICI‐related benefits. Second, the inconsistent pattern of dynamic changes in the immunocyte subpopulation during the therapeutic course also portends divergent drug responses. Third, we additionally analyzed the possible functional relatedness between circulating and tumor‐infiltrating B cells, as well as the TLS in situ, and elucidated that such structure with B cells favored immunotherapy in PLC. Last but not least, an optimal and cost‐effective model was constructed for predicting the efficacy of immunotherapy using non‐invasively available peripheral blood samples, which may be eminently transferable to the clinical practice.

Monocytes belong to mononuclear phagocytes (MNPs) that play important roles in antimicrobial defense and immune regulation. The specific functions of monocytes in the context of tumors have been the subject of extensive debate and research. One area of investigation has focused on a subset of monocytes called monocytic myeloid‐derived suppressor cells (M‐MDSCs), which are distinct from classically activated monocytes.^[^ ^20^ ^]^ Classically inflammatory CD16^−^monocytes infiltrated more in the normal liver compared with liver cancer and highly expressed M1‐macrophage genes involved in inflammatory cytokines,^[^ ^21^ ^]^ suggesting that monocytes may have different functions and characteristics depending on the context. Similar with our CyTOF data, another study showed that a strong predictor for better response to anti‐PD‐1 immunotherapy was the high frequency of CD14^+^CD16^−^HLA‐DR^hi^ monocytes in melanoma^[^ ^22^ ^]^ and non‐small cell lung cancer (NSCLC).^[^ ^23^ ^]^ These results emphasized our discovery that classical monocytes enact as immune modulators during exposure to immunotherapy and may directly sway responsiveness.

T cells were reported to be early responders to cancer immunotherapy in several types of tumors. HLA‐DR is often considered an activation marker for T cells.^[^ ^24^ ^]^ Paradoxically, HLA‐DR^+^CD8^+^T cells we categorized in the context did elevate in peripheral blood from non‐responders before treatment, whereas such clusters in situ tumors may be beneficial for therapy, as also validated by the TCGA external HCC cohort. One possible explanation for this might be that these T cell compartments overexpress the senescent marker CD57 along with a range of immunosuppressive markers, i.e., PD‐1, CTLA4, CD38, and BTLA, suggestive of their replicative deficiency and detrimental impact on respondence of ICIs. Alternatively, the steadily escalating proportion of HLA‐DR^+^CD8^+^T cells in circulation may inevitably lead to resistance against immunotherapy.

Mounting evidence has highlighted the significance of B cells in shaping the tumor microenvironment and influencing immunotherapy outcomes. Studies have shown that B cells expressing high levels of PD‐1 can contribute to a pro‐tumorigenic and chemo‐resistant phenotype in various types of tumors, including HCC.^[^ ^25^, ^26^ ^]^ In contrast, tumor‐infiltrating B cells (TIL‐Bs) play a powerful anti‐tumor role, particularly in mature TLS, where they function as tumor‐specific antibody producers and antigen presenters.^[^ ^27^ ^]^ This hinted that B cells within the tumor microenvironment can have multifaceted interactions with immunotherapy. Our conflicting conclusions drawn about the predictive value of circulating and in situ B cells in immunotherapy efficacy may be attributed to the different roles of B cells in varying contexts. Phenotype analyses also illuminated B‐cell heterogeneity in PLC and recognized a number of B‐cell surface molecules associated with immunotherapy efficacy. Consistent with our results that circulating B cells from non‐responders expressed higher CD45RA and BTLA, Ouyang et al. reported that B cells derived from HCC blood were mainly CD45RA^+^IgD^+^IgM^+^IgG^−^BTLA^+^ and functionally incompetent.^[^ ^28^ ^]^ In this scenario, we preliminarily inquired about the possible roles of the characteristics of B cells in situ, mIHC results demonstrated that there were more B cells in the immunotherapy‐responsive tumors and the majority of B cells were involved in constituting TLS. Furthermore, both of orthotopic murine liver cancer models and in vitro co‐culture experiments demonstrated that B cells in liver cancer microenvironment had synergistic antitumor effects with ICI‐therapy. We postulated that B cell subsets should function differently in situ versus in circulation, the decrease in circulating B cells of responders may be interrelated with the augmented infiltration of tumoral effector B cells, which may exert a concerted anti‐tumor activity with immunotherapy through TLS composition in liver cancer.

A previous study with 10 HCC has linked lower baseline levels of peripheral blood PD‐1^+^B cell and PD‐L1^+^ monocytes to better disease control of ICI,^[^ ^29^ ^]^ and highly activated circulating CD8^+^ T cells during pembrolizumab treatment to clinical benefit^[^ ^30^ ^]^ in HCC. Another research has identified CXCR3^+^CD8^+^ effector memory T (TEM) cells and CD11c^+^ antigen‐presenting cells (APC) in PBMCs associated with response in six paired samples pre‐ and 6 weeks post‐ICI therapy using CyTOF.^[^ ^8^ ^]^ The disaccord of our conclusion may be due to three reasons: 1) Larger numbers of patients were employed in our cohorts; 2) The different design for panels we used in CyTOF and sampling procedure led to an inconsistent emphasis on the presentation of results; 3) The majority of the patients in our cohort were administrated with the combination immunotherapy rather than monotherapy. To better understand the response‐related immune subpopulations and their phenotypes in liver cancer, thorough functional and mechanical studies on the subpopulations identified in this study are warranted.

Given the grave prognosis of advanced PLC and the widespread use of clinical diagnostic criteria for HCC, non‐invasive and prospective surveillance of systemic therapy is crucial for clinical decision‐making. Our predicting model PISIR holds the potential to serve as an effective and minimally invasive biomarker for monitoring early response to ICI‐based systemic therapy in PLC patients. Unlike currently recommended blood‐based tests such as AFP or CEA levels for monitoring tumor progression, PISIR integrates the proportions of specific immune clusters and their characteristic surface molecules prior to treatment. This model shows promise in predicting early immunotherapy efficacy by distinguishing between responders and non‐responders, achieving a competitive AUC (area under the curve) of over 0.9 in all datasets. In spite of this, further investigation involving a larger cohort of prospective patients is warranted to verify the performance characteristics of this blood immune signature biomarker. Once validated, this biomarker can be readily applied in clinical settings.

Integratedly, our results allowed us to discriminate the responders from non‐responders before ICI‐based therapy initiation and tailor the utilization of this treatment to only those patients who are probably to benefit from it. Moreover, we provided conceivable immuno‐targets that could be used to explore novel therapeutic strategies. Nonetheless, there were still limitations and constraints in this study. The antibody panels of CyTOF need careful design and validation, experienced labor is necessary, and both the instrument and the reagents are expensive. These limitations restrained the wide application of CyTOF in clinical practice at present. More ameliorations are needed to permit more hospitals to gain access to a CyTOF machine for use by clinicians. Another limitation was the relatively small sample size of this study, which could induce an misestimation of the differentiation power of PISIR. Another limitation is the relatively small sample size and insufficient homogeneity of the concurrent cohort, which comprised two different pathologic types of liver cancer, potentially leading to misestimation of the ability of PISIR to differentiate. To this end, we plan to conduct a prospective study on a multi‐center level, to verify our current verdict and analyze the underlying biological functions of key immune subpopulations in liver cancer. Nevertheless, these obstacles do not compromise our conclusions, which were established from comprehensive, high‐dimensional analyses of the immune profiles in PBMCs and in situ at different time‐points before and after ICI‐based systemic therapy.

## 4 Experimental Section

### Patients and Sample Characteristics

Patient samples used in this study were obtained from the Cancer Center of Wuhan Union Hospital, with approval from the Hospital Ethics Committee. Written informed consent was obtained from all participants. Between June 2019 and April 2022, a total of 77 patients with unresectable primary liver cancer (71 hepatocellular carcinoma [HCC] patients and six intrahepatic cholangiocarcinoma [ICC] patients with MMR‐proficient tumors) who underwent standard anti‐PD1 immunotherapy‐based systemic therapy were included in the study (Tables S1 and S2, Supporting Information). A subset of 24 patients PBMCs were collected and served as the discovery cohort. For the establishment of the predicting model, the discovery cohort was randomly divided into training and testing cohorts, and an additional 16 patients were enrolled to form the validation cohort. CyTOF analysis was performed on PBMCs obtained from all patients in the three datasets. Furthermore, tumor samples from 18 patients in the cohort were collected prior to ICI‐based therapy and subjected to multiplex fluorescent immunohistochemistry.

The immune checkpoint inhibitors used in the study included camrelizumab (200 mg IV every 21 days), and sintilimab (200 mg IV every 21 days), and the patients receiving combinational therapy were administered Lenvatinib as an anti‐angiogenic drug. Among the 77 enrolled patients, 68 received frontline treatment, while 9 received ICI‐based therapy after Lenvatinib treatment in the second line. Baseline (referred to as “pre‐”) samples and samples obtained after three cycles of ICI‐based therapy (referred to as “post‐”) were collected from the patients at regular intervals. Due to dropouts during therapy, a total of 40 PBMC samples were included in the final analysis. The clinical characteristics of the 24 patients in the discovery cohort and 16 patients in the validation cohort 1 are presented in Table S2 (Supporting Information).

### Clinical Data

Patients were assessed radiographically before the first cycle (baseline) and 12 weeks after ICI exposure (post) to ICI‐based therapy, regardless of dose delays. Disease assessments were collected until radiographic progression or the initiation of subsequent therapy, whichever occurred first. Patients were subsequently followed for survival. Baseline or as well as post‐treatment blood specimens were collected for analyses in the clinical laboratory of Wuhan Union Hospital. Demographic information, including gender, age, and medical history, was recorded for all participants. Radiographic, serous biochemistry and hemagglutination indices were assessed, including chest CT scans, enhanced abdominopelvic magnetic resonance imaging (MRI), blood count, biochemistry test, CRP and virological testing. Baseline samples from 68 patients were available for basic immune profile (5 general immune lineages and cytokines) assessment, and 75 patients for tumor markers, respectively.

The response of PLC patients to ICI‐based treatment was categorized according to the Response Evaluation Criteria in Solid Tumors 1.1 (RECIST v1.1) as complete response (CR), partial response (PR), stable disease (SD), or progressive disease (PD). Patients achieving CR or PR within the first 12 weeks of treatment were classified as responsive (R), while those with SD or PD were classified as non‐responsive (NR).

### Blood Sample Processing and PBMCs Preparation for CyTOF

Whole blood was drawn into BD Vacutainer tubes with EDTA and then processed within 4 h. Five to ten milliliters of blood were transferred to a 50 mL conical tube then an equal volume of PBS was added. 20–40 mL of Ficoll (Sigma) was added to each of the 50 mL tubes and mix the diluted blood samples into them at a ratio of 1:1. The mixture was centrifuged for 25 min at 450 g at room temperature. The top plasma and PBMC layer were separated, and PBMCs were concentrated in the middle layer of the mixed liquid after being centrifuged and washed using phosphate buffer saline (PBS) at 4 °C. Cells were then counted by adding 10 µL of trypan blue to 10 µL of PBMC solution and enumerated on an electronic cell counter (IC100, Countstar). Cells were centrifuged again at 400 g for 5 min at 4 °C then resuspended in commercial cell cryopreserving solution (A1371301, Thermo Fisher) at −80 °C for 24 h, then the frozen samples were transferred to liquid nitrogen. PBMCs were thawed within 12 months for CyTOF analysis.

### Mass‐Tagged Cellular Barcoding, Pre‐Conjugated Antibody Staining, and Mass Cytometry

To minimize the inter‐sample variation, fixed cells from each sample were barcoded using a Cell‐ID 20‐Plex Pd Barcoding Kit (Fluidigm, 201060) consisting of unique combinations of the barcoding reagents 102Pd, 104Pd, 105Pd, 106Pd, 108Pd, and 110Pd, according to the manufacturer's instructions.^[^ ^31^ ^]^ The samples were then washed twice in cell staining buffer, and 20 samples were pooled for antibody surface staining. The pooled leukocytes were incubated in Human TruStain FcX (Fluidigm, 422302) for Fc receptor blocking. The samples were then incubated with both antibody panels for 30 min at room temperature and subsequently washed twice with cell staining buffer. Afterward, samples were stained overnight with 125 nm of Cell‐ID intercalator (Fluidigm, 201067) in fix and perm buffer (Fluidigm, 201192A). Then cells were rinsed twice with cell staining buffer and twice with double distilled water. For mass cytometry acquisition, cells were diluted to 1 million cells/mL in ddH2O comprising 10% EQ four‐element calibration beads (Fluidigm, 201078) and filtrated through FACS tubes with 40 µm filter caps. Samples were uploaded to the mass spectrometer (Fluidigm, Helios) and data were automatically captured, processed, and de‐barcoded as single. FCS documents for each sample. The antibodies used are listed in the Table S3 (Supporting Information).

### CyTOF Data Analysis

CyTOF data were acquired and analyzed as previously described.^[^ ^32^ ^]^ Following Fluidigm's recommendation, FCS files were randomized and normalized using EQ beads signal. After that, the files were concatenated, de‐barcoded, and randomized as instructed by Fluidigm. Then the files were introduced into Cytobank for manual gating of either lymphocytes or T cells using the concatenation tool. Lymphocyte or T‐cell events were exported as.fcs files and loaded to R for analysis downstream. For deeper analysis, high‐dimensional data were visualized by the t‐SNE algorithm into 2D data (default settings: seed = 42, tSNE complexity = 30, tSNE maximum number of iterations = 1000) in the R package “cytofkit”. Clustering analysis was executed using Phenograph applied to all samples run (nearest neighbors K = 30, Euclidean distance). T‐SNE maps were produced by the cytofkitShinyAPP interactive interface. Heatmaps and unsupervised clustering were visualized by the R pheatmap package. Other data were displayed with the ggplot2 R package or Graphpad Prism 9.

### Survival Analysis

Survival analyses for PFS and OS were carried out via the two‐sided log‐rank test by the GraphPad Prism 9. It compared PFS and OS in responders versus non‐responders, and patients with high‐levels versus low‐levels of a certain factor. High‐ or low‐levels of a certain factor were defined, respectively, by the values of the factor ≥ median value or values of the factor < median value across the cohorts.

### Multiplex Fluorescent Immunohistochemistry (mIHC)

Eighteen paired tumors and the normal formalin‐fixed paraffin‐embedded tissue from PLC patients were stained with CD8, CD19, HLA‐DR, CD20, and DAPI by Opal Polaris 7‐Color Automation IHC Kit (AKOYA BIOSCIENCES, NEL871001KT) according to the instruction and scanned using a PhenoCycler‐Fusion System Microscope (Akoya Biosciences). After serial dewaxing and rehydration processes, slides were fixed with 10% neutral buffered formaldehyde for 20 min. Heat‐induced antigen retrieval was performed using AR Buffer (Perkin–Elmer kit) in a microwave oven for 30 min. Slides were rinsed with ddH_2_O and TBST once, blocked using Blocking Buffer (Perkin–Elmer kit), and stained with antibodies according to the user manual. DAPI was used as a nuclear counterstain. The digital images were analyzed with QuPath v0.3.0. For example, based on the above results, as well as single‐cell transcriptome analysis, the expression levels of PD‐1, CD19, CD20, CD8, and HLA‐DR were used to evaluate the enrichment of TLS (CD20^+^B cells co‐localized with CD8^+^T) and related immune subpopulations (HLA‐DR^+^PD‐1^+^CD8^+^ T cells and CD19^+^B cells^[^ ^33^ ^]^). The ratio of each cell subpopulation to the total cells was calculated and applied for comparison. Details about the antibodies used were listed in Table S3 (Supporting Information).

### Model Development

After comparison of the differential expressed markers, two parameters with adjust p values<0.05 (FDR adjustments) and the former identified differentially enriched immune subpopulations were integrated as risk factors: 1) percentage of classical monocytes (Classical_Monocyte%), 2) percentage of B cells (Bcell%), 3) percentage of HLA‐DR^+^CD8^+^T cells (HLA‐DR^+^CD8^+^T%), 4) expression level of CD45 in NK cells [NK (CD45exp)], 5) expression level of CD279 on T cells [Tcell (CD279exp)]. These five variables were used as the input of the machine learning models. It conducted generalized linear regression models (denote as PISIR) of the binomial family with a log link to estimate the likelihood of selected exposure variables and ICI‐based therapy response. The model was trained on the training cohort (n = 12) of the discovery cohort and validated twice on the testing cohort (n = 11) and validation cohort1 (n = 16).

### Statistical Analysis

Complete statistics details of the experiments, data analysis and presentation were available in the figure legends or the manuscript above. Statistical analyses were performed using GraphPad Prism version 9 (Graphpad Software, San Diego, CA, USA) and R software. Comparisons of categorical variables were assessed by chi‐square test or Fisher's exact test, as appropriate. As noted in the legends, the Wilcoxon rank sum test, paired Wilcoxon signed rank test, and Student t‐test were adopted for quantitative variables, and P values ≤0.05 were considered statistically significant. For multiple comparison, p values were adjusted by the Benjamini–Hochberg (BH) method for multiple comparisons. The Pearson correlation test was utilized to test for significant correlations between quantitative variables. Univariate and multivariate Cox proportional hazards regression models and Kaplan–Meier curves (log‐rank test) were used for progression‐free and overall survival analysis.

### Ethics Approval and Consent to Participate

This study was approved by the Wuhan Union Hospital Research Ethics Committee and the Laboratory Animal Ethics Committee, Tongji Medical College, Huazhong University of Science and Technology. Written informed consent was obtained from each patient

## Conflict of Interest

The authors declare no conflict of interest.

## Author Contributions

J.X., S.Y., S.‐S.Z., J.F. contributed equally to this work. J.X. constructed the clinical sample cohort. S.Y., C.S., and Z.W. conducted CyTOF‐related experiments, did the data analysis of CyTOF. S.Z., Z.W., Z.F., Y.Y., and J.T. processed the clinical PBMC samples. J.X. and J.T. wrote the manuscript, did the other experiments. P.L. and X.Q. assisted with clinical data analysis. J.X. and T.Z. organized the figures. S.Z., Z.W., Z.F., and Y.Y. collected the clinical information. J.F. and D.Z. collected the FFPE samples, relevant patients’ information. L.C., G.W., H.W., and J.T. designed the research, supervised the study, guided the discussion, are responsible for revising the manuscript.

## Supporting information



Supporting Information

Supporting Information

## Data Availability

The data that support the findings of this study are available from the corresponding author upon reasonable request.

## References

[1] J. D. Yang , P. Hainaut , G. J. Gores , A. Amadou , A. Plymoth , L. R. Roberts , Nat. Rev. Gastroenterol. Hepatol. 2019, 16, 589. 31439937 10.1038/s41575-019-0186-y PMC6813818

[2] M. Baretti , A. K. Kim , R. A. Anders , Cancer Cell 2022, 40, 252. 35290785 10.1016/j.ccell.2022.02.017 PMC9844534

[3] R. S. Finn , M. Ikeda , A. X. Zhu , M. W. Sung , A. D. Baron , M. Kudo , T. Okusaka , M. Kobayashi , H. Kumada , S. Kaneko , M. Pracht , K. Mamontov , T. Meyer , T. Kubota , C. E. Dutcus , K. Saito , A. B. Siegel , L. Dubrovsky , K. Mody , J. M. Llovet , J. Clin. Oncol. 2020, 38, 2960. 32716739 10.1200/JCO.20.00808 PMC7479760

[4] A. X. Zhu , A. R. Abbas , M. R. de Galarreta , Y. Guan , S. Lu , H. Koeppen , W. Zhang , C. H. Hsu , A. R. He , B. Y. Ryoo , T. Yau , A. O. Kaseb , A. M. Burgoyne , F. Dayyani , J. Spahn , W. Verret , R. S. Finn , H. C. Toh , A. Lujambio , Y. Wang , Nat. Med. 2022, 28, 1599. 35739268 10.1038/s41591-022-01868-2

[5] D.‐Y. Oh , A. R. He , S. Qin , L.‐T. Chen , T. Okusaka , A. Vogel , J. W. Kim , T. Suksombooncharoen , M. A. Lee , M. Kitano , H. A. Burris Iii , M. Bouattour , S. Tanasanvimon , R. Zaucha , A. Avallone , J. Cundom , N. Rokutanda , J. Xiong , G. Cohen , J. W. Valle , J. Clin. Oncol. 2022, 40, 378.

[6] L. Rimassa , R. S. Finn , B. Sangro , J. Hepatol. 2023, 79, 506. 36933770 10.1016/j.jhep.2023.03.003

[7] J. J. Havel , D. Chowell , T. A. Chan , Nat. Rev. Cancer 2019, 19, 133. 30755690 10.1038/s41568-019-0116-x PMC6705396

[8] S. Chuah , J. Lee , Y. Song , H. D. Kim , M. Wasser , N. A. Kaya , K. Bang , Y. J. Lee , S. H. Jeon , S. Suthen , S. A'Azman , G. Gien , C. J. Lim , C. Chua , S. N. Hazirah , H. K. Lee , J. Q. Lim , T. K. H. Lim , J. Yeong , J. Chen , E. C. Shin , S. Albani , W. Zhai , C. Yoo , H. Liu , S. P. Choo , D. Tai , V. Chew , J. Hepatol. 2022, 77, 683. 35430299 10.1016/j.jhep.2022.03.039

[9] M. Pallozzi , N. Di Tommaso , V. Maccauro , F. Santopaolo , A. Gasbarrini , F. R. Ponziani , M. Pompili , Cancers 2022, 14, 4631. 36230554 10.3390/cancers14194631 PMC9559710

[10] B. Scheiner , K. Pomej , M. M. Kirstein , F. Hucke , F. Finkelmeier , O. Waidmann , V. Himmelsbach , K. Schulze , J. von Felden , T. W. Frundt , M. Stadler , H. Heinzl , K. Shmanko , S. Spahn , P. Radu , A. R. Siebenhuner , J. C. Mertens , N. N. Rahbari , F. Kutting , D. T. Waldschmidt , M. P. Ebert , A. Teufel , S. De Dosso , D. J. Pinato , T. Pressiani , T. Meischl , L. Balcar , C. Muller , M. Mandorfer , T. Reiberger , et al., J. Hepatol. 2022, 76, 353. 34648895 10.1016/j.jhep.2021.09.035

[11] S. Kleffel , C. Posch , S. R. Barthel , H. Mueller , C. Schlapbach , E. Guenova , C. P. Elco , N. Lee , V. R. Juneja , Q. Zhan , C. G. Lian , R. Thomi , W. Hoetzenecker , A. Cozzio , R. Dummer , M. C. Mihm Jr. , K. T. Flaherty , M. H. Frank , G. F. Murphy , A. H. Sharpe , T. S. Kupper , T. Schatton , Cell 2015, 162, 1242. 26359984 10.1016/j.cell.2015.08.052 PMC4700833

[12] K. Ma , L. Sun , M. Shen , X. Zhang , Z. Xiao , J. Wang , X. Liu , K. Jiang , F. Xiao‐Feng Qin , F. Guo , B. Zhang , L. Zhang , iScience 2022, 25, 104347. 35602958 10.1016/j.isci.2022.104347 PMC9117873

[13] Z. Chen , Z. Ji , S. F. Ngiow , S. Manne , Z. Cai , A. C. Huang , J. Johnson , R. P. Staupe , B. Bengsch , C. Xu , S. Yu , M. Kurachi , R. S. Herati , L. A. Vella , A. E. Baxter , J. E. Wu , O. Khan , J. C. Beltra , J. R. Giles , E. Stelekati , L. M. McLane , C. W. Lau , X. Yang , S. L. Berger , G. Vahedi , H. Ji , E. J. Wherry , Immunity 2019, 51, 840. 31606264 10.1016/j.immuni.2019.09.013 PMC6943829

[14] A. Larbi , T. Fulop , Cytometry A 2014, 85, 25. 24124072 10.1002/cyto.a.22351

[15] J. Hang , J. Huang , S. Zhou , L. Wu , Y. Zhu , L. Zhu , H. Zhou , K. Xu , H. Jiang , X. Yang , Cancer Med. 2019, 8, 1326. 30767430 10.1002/cam4.1988 PMC6434335

[16] E. Braakman , J. G. van de Winkel , B. A. van Krimpen , M. Jansze , R. L. Bolhuis , Cell. Immunol. 1992, 143, 97. 1377991 10.1016/0008-8749(92)90008-d

[17] S. Kadomoto , K. Izumi , A. Mizokami , Int. J. Mol. Sci. 2020, 21, 5186. 32707869 10.3390/ijms21155186 PMC7432448

[18] A. Martinez‐Sabadell , E. J. Arenas , J. Arribas , Clin. Cancer Res. 2022, 28, 1243. 34785585 10.1158/1078-0432.CCR-21-3226

[19] Y. Hailemichael , D. H. Johnson , N. Abdel‐Wahab , W. C. Foo , S. E. Bentebibel , M. Daher , C. Haymaker , K. Wani , C. Saberian , D. Ogata , S. T. Kim , R. Nurieva , A. J. Lazar , H. Abu‐Sbeih , F. Fa'ak , A. Mathew , Y. Wang , A. Falohun , V. Trinh , C. Zobniw , C. Spillson , J. K. Burks , M. Awiwi , K. Elsayes , L. S. Soto , B. D. Melendez , M. A. Davies , J. Wargo , J. Curry , C. Yee , et al., Cancer Cell 2022, 40, 509. 35537412 10.1016/j.ccell.2022.04.004 PMC9221568

[20] L. van Vlerken‐Ysla , Y. Y. Tyurina , V. E. Kagan , D. I. Gabrilovich , Cancer Cell 2023, 41, 490. 36868224 10.1016/j.ccell.2023.02.009 PMC10023509

[21] K. Mulder , A. A. Patel , W. T. Kong , C. Piot , E. Halitzki , G. Dunsmore , S. Khalilnezhad , S. E. Irac , A. Dubuisson , M. Chevrier , X. M. Zhang , J. K. C. Tam , T. K. H. Lim , R. M. M. Wong , R. Pai , A. I. S. Khalil , P. K. H. Chow , S. Z. Wu , G. Al‐Eryani , D. Roden , A. Swarbrick , J. K. Y. Chan , S. Albani , L. Derosa , L. Zitvogel , A. Sharma , J. Chen , A. Silvin , A. Bertoletti , C. Bleriot , Immunity 2021, 54, 1883. 34331874 10.1016/j.immuni.2021.07.007

[22] C. Krieg , M. Nowicka , S. Guglietta , S. Schindler , F. J. Hartmann , L. M. Weber , R. Dummer , M. D. Robinson , M. P. Levesque , B. Becher , Nat. Med. 2018, 24, 144. 29309059 10.1038/nm.4466

[23] P. Rochigneux , A. Lisberg , A. Garcia , S. Granjeaud , A. Madroszyk , S. Fattori , A. Goncalves , R. Devillier , P. Maby , N. Salem , L. Gorvel , B. Chanez , J. Gukasyan , J. Carroll , J. Goldman , A. S. Chretien , D. Olive , E. B. Garon , Clin. Cancer Res. 2022, 28, 5136. 36166003 10.1158/1078-0432.CCR-22-1386 PMC10085054

[24] M. K. Rahim , T. L. H. Okholm , K. B. Jones , E. E. McCarthy , C. C. Liu , J. L. Yee , S. J. Tamaki , D. M. Marquez , I. Tenvooren , K. Wai , A. Cheung , B. R. Davidson , V. Johri , B. Samad , W. E. O'Gorman , M. F. Krummel , A. van Zante , A. J. Combes , M. Angelo , L. Fong , A. P. Algazi , P. Ha , M. H. Spitzer , Cell 2023, 186, 1127. 36931243 10.1016/j.cell.2023.02.021 PMC10348701

[25] X. Xiao , X. M. Lao , M. M. Chen , R. X. Liu , Y. Wei , F. Z. Ouyang , D. P. Chen , X. Y. Zhao , Q. Zhao , X. F. Li , C. L. Liu , L. Zheng , D. M. Kuang , Cancer Discov. 2016, 6, 546. 26928313 10.1158/2159-8290.CD-15-1408

[26] S. Shalapour , J. Font‐Burgada , G. Di Caro , Z. Zhong , E. Sanchez‐Lopez , D. Dhar , G. Willimsky , M. Ammirante , A. Strasner , D. E. Hansel , C. Jamieson , C. J. Kane , T. Klatte , P. Birner , L. Kenner , M. Karin , Nature 2015, 521, 94. 25924065 10.1038/nature14395 PMC4501632

[27] C. M. Laumont , A. C. Banville , M. Gilardi , D. P. Hollern , B. H. Nelson , Nat. Rev. Cancer 2022, 22, 414. 35393541 10.1038/s41568-022-00466-1 PMC9678336

[28] F. Z. Ouyang , R. Q. Wu , Y. Wei , R. X. Liu , D. Yang , X. Xiao , L. Zheng , B. Li , X. M. Lao , D. M. Kuang , Nat. Commun. 2016, 7, 13453. 27853178 10.1038/ncomms13453 PMC5118541

[29] Y. P. Hung , Y. Y. Shao , J. M. Lee , C. Hsu , C. H. Hsu , M. H. Yang , Y. Chao , J. Chin. Med. Assoc. 2021, 84, 144. 33433132 10.1097/JCMA.0000000000000477 PMC12965983

[30] J. Y. Hong , H. J. Cho , J. K. Sa , X. Liu , S. Y. Ha , T. Lee , H. Kim , W. Kang , D. H. Sinn , G. Y. Gwak , M. S. Choi , J. H. Lee , K. C. Koh , S. W. Paik , H. C. Park , T. W. Kang , H. Rhim , S. J. Lee , R. Cristescu , J. Lee , Y. H. Paik , H. Y. Lim , Genome Med. 2022, 14, 1. 34986867 10.1186/s13073-021-00995-8 PMC8734300

[31] E. R. Zunder , R. Finck , G. K. Behbehani , A. D. Amir el , S. Krishnaswamy , V. D. Gonzalez , C. G. Lorang , Z. Bjornson , M. H. Spitzer , B. Bodenmiller , W. J. Fantl , D. Pe'er , G. P. Nolan , Nat. Protoc. 2015, 10, 316. 25612231 10.1038/nprot.2015.020 PMC4347881

[32] S. Yang , L. Qian , Z. Li , Y. Li , J. Bai , B. Zheng , K. Chen , X. Qiu , G. Cai , S. Wang , H. Huang , J. Wu , Y. Zhu , Q. Zhangyang , L. Feng , T. Wu , R. Wu , A. Yang , K. Wang , R. Wang , Y. Zhang , Y. Zhao , W. Wang , J. Bao , S. Shen , J. Hu , X. Wu , T. Zhou , Z. Meng , W. Liu , et al., Gastroenterology 2023, 164, 407. 36574521 10.1053/j.gastro.2022.11.029

[33] C. Zheng , L. Zheng , J. K. Yoo , H. Guo , Y. Zhang , X. Guo , B. Kang , R. Hu , J. Y. Huang , Q. Zhang , Z. Liu , M. Dong , X. Hu , W. Ouyang , J. Peng , Z. Zhang , Cell 2017, 169, 1342. 28622514 10.1016/j.cell.2017.05.035

