# Supplementary material for: Deciphering the Multifaceted Immune Landscape of Unresectable Primary Liver Cancer to Predict Immunotherapy Response
Source: Adv Sci (Weinh). 2024 Oct 28;11(47):2309631. doi: 10.1002/advs.202309631 (PMC11653612; doi:10.1002/advs.202309631)
Supplement: Supplementary file 1 — Supporting Information [file ADVS-11-2309631-s001.pdf]

ADVANCED SCIENCE
Open Access

Supporting Information

for *Adv. Sci.*, DOI 10.1002/advs.202309631

Deciphering the Multifaceted Immune Landscape of Unresectable Primary Liver Cancer to Predict Immunotherapy Response

Jun Xue, Shuai Yang, Si-Si Zhang, Jun Fan, Zi-Long Wu, Cheng-Jun Sui, Yong-Qiang Yang, Jin-Feng Zhang, Pian Liu, De-Jun Zhang, Xin-Yao Qiu, Tao Zhang, Lei Chen, Gang Wu*, Hong-Yang Wang* and Jing Tang**

Deciphering the Multifaceted Immune Landscape of unresectable Primary Liver Cancer to Predict Immunotherapy Response

Jun Xue, Shuai Yang, Si-Si Zhang, Jun Fan, Zi-Long Wu, Cheng-Jun Sui, Yong-Qiang Yang, Jin-Feng Zhang, Pian Liu, De-Jun Zhang, Xin-Yao Qiu, Tao Zhang, Lei Chen, Gang Wu, Hong-Yang Wang, Jing Tang

Supplementary figure legends

Figure S1 (A) Representative MRI scans displaying target lesions in patient #51 with partial response (PR) and patient #46 with progressive disease (PD). (B) Kaplan–Meier plots illustrating progression-free survival (PFS) and overall survival (OS) in our study involving all biologically independent subjects with follow-up survival data (n=75). The patients from the N (responders) and NR (non-responders) groups were stratified. Hazard ratio (HR) and two-sided log-likelihood p-values are presented, with 95% confidence intervals (CI) indicated in parentheses. (C) Gating strategy employed in panel 1 to identify T cells, B cells, natural killer (NK) cells, monocytes, and macrophages. (D) Gating strategy employed in panel 2 to identify T cells, CD16+ NK cells, monocytes, B cells, and CD16+ myeloid dendritic cells (mDCs). (E) Correlation analysis revealing the normalized expression of overlapping markers between panel 1 and panel 2. (F) Correlation analysis examining the percentages of B cells, monocytes, and T cells between panel 1 and panel 2. (G) Correlation analysis between normalized expression of each marker from two repeated CyTOF test samples obtained during two separate runs.

Figure S2 (A) Representative examples of t-SNE plots illustrating the normalized marker expression from all samples in Panel 1. (B) Heatmap displaying the mean

expression level of all 32 markers in 31 subclusters and 9 classified cell types. (C) Cell percentages of subclusters including B cells (C21, C24, C5, C7, and C9) and classical monocytes (C1 and C15) are plotted and compared between responders (R) and non-responders (NR) post-treatment. (D) Cell percentages of macrophages, myeloid dendritic cells (mDC), natural killer (NK) cells, T cells, plasmacytoid dendritic cells (pDC), and non-classical monocytes (NCM) are plotted and compared between responders (R) and non-responders (NR) both pre-treatment and post-treatment. Wilcoxon rank-sum test was used to identify significant differences. (E) Dynamic changes in the percentage of mDC before (pre) and after (post) ICI-based therapy are depicted in patient-matched PBMCs. The left panel represents data from all 16 patients, the middle panel focuses on 8 responders, and the right panel focuses on 8 non-responders. Paired Wilcoxon signed rank test was used to identify significant differences. The line and box represent the mean and upper and lower quartiles, respectively.

Figure S3 Violin plots are presented to depict the normalized median expression of the differentially expressed markers from panel 1 in each cell of the indicated immune cell subtypes. A two-sided Wilcoxon rank-sum test was conducted to compare the expression levels of CD38, CD64, MRC-1, and Siglec-1 between the specified cell types from the responder (R) and non-responder (NR) groups. Only significant p-values are shown (*$p < 0.05$, **$p < 0.01$, ***$p < 0.001$).

Figure S4 (A) Heatmap displaying the mean expression level of all 34 markers in 25 clusters, 15 cell subpopulations, and 6 cell subtypes. The heatmap represents the average expression values of the markers, with color indicating the expression intensity. The dendrograms depict hierarchical clustering of the markers and subpopulations.

(B) t-SNE plots of panel 2 showing the identification of 10 classical T cell subpopulations and 5 non-T cell subpopulations in PBMCs using the phenograph clustering method. The plots visualize the spatial distribution of cells in a reduced-dimensional space, with different colors representing distinct subpopulations. (C) Representative examples of t-SNE plots illustrating the normalized marker expression

from all samples in Panel 2. (D) Violin plots are presented to depict the normalized median expression of CD4 and CD9 in $CD9^{+}CD4^{+}$(T3 and T9), $CD9^{-}CD4^{+}$ (T5, T8, and T14) T cell subclusters, and CD8 and HLA-DR in HLA-$DR^{+}CD8^{+}$ (T20 and T21) and HLA-$DR^{-}CD8^{+}$ (T4, T7 and T24) T cell subclusters, respectively. The plots display the distribution of expression values, with the width of the violin indicating the density of cells at different expression levels. (E) Frequencies of immune subsets identified by panel 2 are depicted for each sample, with the left panel representing pre-treatment samples and the right panel representing post-treatment samples. The analysis comprised samples of 8 responders (Rs) and 15 non-responders (NRs) in the pre-treatment cohort and 9 Rs and 8 NRs in the post-treatment cohort, respectively. (F) Dynamic changes in the percentage of HLA-$DR^{+}CD8^{+}$ T cell subclusters (T20 and T21) pre- and post-ICI-based therapy are depicted in patient-matched PBMCs from 8 responders (left panel) and 8 non-responders (right panel). The line graphs show the changes in the percentage of cells in each subcluster over time for responders and non-responders. The line and box represent the mean and upper and lower quartiles, respectively. (G) Cell percentages of cluster T12 (right) and T13 (left) are plotted and compared between responders (Rs) and non-responders (NRs) both pre-treatment (left) and post-treatment (right). (H) Dynamic changes in the percentage of CTLA-4^{-} monocytes (T12) pre- and post-ICI-based therapy are depicted in patient-matched PBMCs from all patients (left panel), responders (middle panel) and non-responders (right panel). The line graphs show the changes in the percentage of cells in each subcluster over time for responders and non-responders. The line and box represent the mean and upper and lower quartiles, respectively. Wilcoxon rank-sum test and paired Wilcoxon signed rank test (for paired data) were used to identify significant differences.

Figure S5 (A) Heatmap displaying the mean expression level of all 34 markers in 18 T-cell clusters and 4 cell subpopulations. The heatmap represents the average expression values of the markers, with color indicating the expression intensity. The dendrograms depict hierarchical clustering of the markers and subpopulations. (B) Cell percentages of $CD45^{+}CD57^{-}CD8^{+}$ T cells (TC10) are plotted and compared between

responders (Rs) and non-responders (NRs) pre-treatment. (C-D) Dynamic changes in the percentage of one HLA-DR^{+}CD8^{+} T cell subcluster (C, TC12) pre- and post-ICI-based therapy are depicted in patient-matched PBMCs from all patients in discovery cohort. (D) Dynamic changes in the percentage of circulating CD8^{+}Tcm (TC4) pre- and post-ICI-based therapy are depicted in patient-matched PBMCs from all patients (left panel), responders (middle panel) and non-responders (right panel). The line graphs show the changes in the percentage of cells in each subcluster over time for responders and non-responders. The line and box represent the mean and upper and lower quartiles, respectively. Wilcoxon rank-sum test and paired Wilcoxon signed rank test (for paired data) were used to identify significant differences.

Figure S6 (A) Heatmap showing the scaled normalized expression for the top 10 markers with the most differential expression between responders and non-responders before (upper panel) and 12 weeks after (lower panel) initiation of therapy. The color scale represents relative marker expression, with yellow indicating overexpression and blue indicating under-expression. Median expression was calculated on single, live CD45+ cells obtained from thawed PBMC samples. Dendrograms display hierarchical clustering of markers (rows) and samples (columns) using Euclidean distance. Bars at the top represent individual samples, with responders shown in orange and non-responders in grey. Each column represents a patient sample, with a total of 23 patients before treatment and 17 patients after treatment. R denotes responders, and NR denotes non-responders. (B) Heatmap showing the scaled normalized proportion of immune cell subpopulations identified in panel 2, for the top 10 cell subpopulations with the most differential enrichment between responders and non-responders before (left panel) and 12 weeks after (right panel) initiation of therapy. Median proportions were calculated based on single, live CD45+ cells derived from thawed PBMC samples. (C) Heatmap showing the scaled normalized expression of markers on the indicated cell subpopulations in panel 2, for the top 10 cell-specific surface markers with the most differential expression between responders and non-responders before (left panel) and 12 weeks after (right panel) initiation of therapy. Median expression was calculated on

indicated single cell subpopulations classified from panel 2. (D) Violin plots are presented to depict the normalized median expression of the differentially expressed markers from panel 1 in each cell of the indicated immune cell subtypes. A two-sided Wilcoxon rank-sum test was conducted to compare the expression levels of CD38, CD64, MRC-1, and Siglec-1 between the specified cell types from the responder (R) and non-responder (NR) groups. Only significant p-values are shown (*$p < 0.05$, **$p < 0.01$, ***$p < 0.001$). (E) Clustering tree for the relationships among all 34 surface markers from panel 2 in patients classified as responders (R) and non-responders (NR) before ICI-based therapy.

Figure S7 (A) The remained serum level of cytokines is plotted and compared between responders (R) and non-responders (NR) pre-treatment. The Kruskal-Wallis test was used to identify significant differences. The analysis included a total of 68 patients for whom serum levels of common cytokines were tested in the clinical laboratory. (B) Association between Intratumoral PD-L1 status and therapeutic response, respectively. Two-tailed chi-square test was used to determine statistical significance between the groups; ns, not significantly. (C-F) Kaplan-Meier survival analysis for OS and PFS of PLC patients with high or low densities of intratumoral PD-L1 status (C), B cells (D), TLS (E), and HLA-DR^{+}CD8^{+} T cells (F, HT represented HLA-DR^{+}CD8^{+}T cells). PD-L1 status was defined as positive and negative, medians of other continuous parameters were defined as the cutoff value. The analysis comprised 18 patients who were included based on the availability of mIHC data and follow-up survival data. (G) TIMER2.0 and CIBERSORT (http://timer.cistrome.org/) were used for investigating the associations between CD8^{+}T infiltrates, HLA-DRA expression, and clinical prognosis. Kaplan-Meier estimates of OS in patients in liver cancer cohort from The Cancer Genome Atlas (TCGA-LIHC, n = 340 biologically independent subjects). Stratified hazard ratios and likelihood p values for death are reported.

Figure S8 (A) Flow chart for the construction of an orthotopic liver cancer model, and the indicated drug treatment model. (B) In vivo imaging of tumor-bearing mice with the indicated treatment using luciferase (left panel); and quantification of the

fluorescence signals in vivo experiments (right panel). All the fluorescent intensities were measured in counts/energy/area. (C) Wet liver weights of tumor-bearing mice with the indicated treatment. (D) Gating strategies for B cells and $CD8^{+}$T cells with the indicated surface markers. (E) Representative images of $CD8^{+}$T-cell and $B220^{+}$B-cells infiltration by immunohistochemistry using orthotopic liver tumor samples collected 40 days after cell injection. Scale bar = 100μm. (F) Percentages of $CD4^{-}CD8^{+}$T cells in tumor infiltrating $CD45^{+}$ cells of Hepa1-6 liver tumor (left panel); Percentages of PD-1^{+}/Ki-67^{+}/IFN-γ^{+}/$Perforin^{+}$ $CD8^{+}$T in total tumor infiltrating CD4-$CD8^{+}$T cells of Hepa1-6 liver tumor; (G-H) Co-culture assay of tumor-infiltrating B cells, $CD3^{+}$T cells and Hepa1-6 cells obtained from the liver tumor-bearing mice; (G) Crystal violet staining of the remained tumor cells after co-culture and indicated treatment for 48 hours. (H) Percentages of PD-1^{+}/Ki-67^{+}/IFN-γ^{+}/$Perforin^{+}$ $CD8^{+}$T in total $CD4^{-}CD8^{+}$T cells of with the indicated co-culture for 48 hours; C, Hepa1-6 tumor cells; T, $CD3^{+}$T cells; B, B cells; The line and box represent the mean and upper and lower quartiles (n = 4), respectively. Wilcoxon rank-sum tests were used to identify significant differences. *$p < 0.05$, **$p < 0.01$, ***$p < 0.001$, ****$p < 0.0001$.

Supplementary Materials and Methods

Antibody panels designing for CyTOF

A panel for the canonical immune cell population consisted of 32 pre-conjugated antibodies (Panel 1), and a T cell-centered panel (Panel 2) composed of 34 pre-conjugated antibodies, both designed and purchased by Fluidigm (Table S3). Panel 1 is designed based on the commercial 15-parameter panel-Maxpar® Human Monocyte/Macrophage Phenotyping Panel Kit (Fluidigm, San Francisco, CA, USA), which is more focused on the detailed analysis of myeloid cells such as monocytes and macrophages. We have extended the total number of antibodies to 32, including PD-1, OX40, PD-L1, and etc., enabling a more systematic identification and evaluation of the function of the myeloid lineages as well as B cells and NK cells.

Panel 2 was updated from the 16-parameter panel Maxpar Human T-Cell

Phenotyping Panel Kit (Fluidigm, San Francisco, CA, USA) that allow for the identification of major T-cell subsets including naive, central memory, effector, and effector memory $CD4^+$ and $CD8^+$ cells, and also classify the activation and homing status of these subtypes. Based on this kit, we have integrated CD134(OX40), CD137(4-1BB), CD152(CTLA-4), CD279 (PD-1), and other markers that refer to T-cell activation, suppression or exhaustion, bringing the total number of antibodies to 34. This custom-designed panel enabled us to accomplish a comprehensive and in-depth evaluation of T-cell functions simultaneously.

Establishment and drug treatment of the orthotopic transplantation tumor model

Six-week-old male C57BL/6 mice were purchased from Vital River Laboratory (Beijing, China). All of the animal experiments were approved by the Medical Ethics Committee of Tongji Medical College, Huazhong University of Science and Technology, and the experiments were performed according to the guidelines for the care and use of laboratory animals.

Orthotopic liver cancer models were established by injecting 1×10^6 Hepa1-6-luc HCC cells into the subcapsular area of the left liver lobe of male C57BL/6 mice, as previously described[1]. Mice were randomized to different treatment groups after tumor implantation (n=4). Treatment with anti-mouse PD-1 antibody (BE0146, Bio X Cell, West Lebanon, NH, USA), or isotype control antibody, 0.2 mg per dose, was given on the indicated days (Figure S8A) after tumor implantation by intraperitoneal injection.

To evaluate the roles of B cells in the antitumor efficacy of anti-PD-1 antibodies, B cells were depleted by intraperitoneal injection of anti-CD20 antibody or isotype control antibody (BP0356, Bio X Cell, West Lebanon, NH, USA), respectively. The dosage of antibody treatment was 0.2 mg on day 0, 10, 20, and 30 after tumor implantation. Depletion of B cells was confirmed by flow cytometry as previously described[2]. In vivo imaging assay was conducted and live weights were measured after 40 days of tumor implantation. At the end of treatment, tumor-infiltrating and circulating B cells, $CD3^+$T cells, splenic $CD3^+$T cells was extracted as described[3], and formalin-fixed paraffin-embedded (FFPE) tumor samples were collected for analysis of

tumor-infiltrating lymphocytes (TILs).

Co-culture of B, T, and tumor cells

2.5× 10^5 Hepa1-6 HCC cells were plated in a 24-well plate in triplicate per condition. A co-culture system that included the sorted TIL-B cell, CD 3+T cell, and tumor cells previously described has been established[4]. The CD40-L (1 μg/mL) activated B-cells, pre-stimulated T cells with anti-CD3 and anti-CD28 antibodies, and tumor cells were cultured in a 3:3:1 ratio for 48 hours, with or without anti-PD-1(20μg/ml). Then T cells and B cells were harvest for testing of functional marker by Flow Cytometry, and the adherent tumor cells were fixed with methanol and stained with 0.1% crystal violet (Sigma-Aldrich, St. Louis, MO, USA) in PBS for 15 min. The stained tumor cells per well were photographed and quantified in triplicate for each treatment group.

Flow cytometry

Antibodies used to measure the expression of cell surface markers using flow cytometry included CD3, CD4, CD8, CD45, Ki-67, IFN-γ, Perforin, CD45R/B220 and appropriate isotype controls. PD-1 antibody and anti-CD20 antibody were both purchased from BioXCell®. Stained cells were analyzed using a BD FACS CELEASTA flow cytometer (BD Biosciences) with FlowJo software (TreeStar, Ashland, USA).

Immunohistochemistry (IHC)

The tissues were immersed in 4% paraformaldehyde for 4h and transferred to 70% ethanol. Individual lodes of liver biopsy were placed in processing cassettes, dehydrated through a serial alcohol gradient, and embedded in paraffin wax blocks. Before immunostaining, 5um-thick- tissue sections were dewaxed in xylene, rehydrated through decreasing concentrations of ethanol, and washed in PBS. The sections were placed in 3% H_2O_2 methanol solution at room temperature for 10 minutes for inactivation of endogenous hydrogen peroxide. Antigen were unmasked by microwaving sections in 10mmol/L citrate buffer (pH 6.0) for 15 min, and immunostaining was undertaken using Avidin: Biotinylated enzyme Complex (ABC)

method with antibodies against mouse CD8 and B220 (see details in Table S3). After being washed, the slices were incubated with the appropriate biotin-conjugated secondary antibody for 1 h at room temperature and then color development was performed using 3,3-diaminobenzidine tetrahydrochloride (DAB) as a chromogen. Sections were counterstained using Gill-2 hematoxylin (Thermo-Shandon, Pittsburgh, PA). After staining, sections were dehydrated through increasing concentrations of ethanol and xylene. IHC staining images were analyzed using Aperio ImageScope v12.32.8013(Lecia Biosystems).

Reference:

1. Chang, C. J.,Chen, Y. H.,Huang, K. W.,Cheng, H. W.,Chan, S. F.,Tai, K. F.,Hwang, L. H., *Hepatology* **2007,** *45*, 746.

2. Kishihara, K.,Penninger, J.,Wallace, V. A.,Kundig, T. M.,Kawai, K.,Wakeham, A.,Timms, E.,Pfeffer, K.,Ohashi, P. S.,Thomas, M. L.,et al., *Cell* **1993,** *74*, 143.

3. Xiao, X.,Lao, X. M.,Chen, M. M.,Liu, R. X.,Wei, Y.,Ouyang, F. Z.,Chen, D. P.,Zhao, X. Y.,Zhao, Q.,Li, X. F.,Liu, C. L.,Zheng, L.,Kuang, D. M., *Cancer Discov* **2016,** *6*, 546.

4. Zhou, X.,Su, Y. X.,Lao, X. M.,Liang, Y. J.,Liao, G. Q., *Oral Oncol* **2016,** *53*, 27.

Figure S1

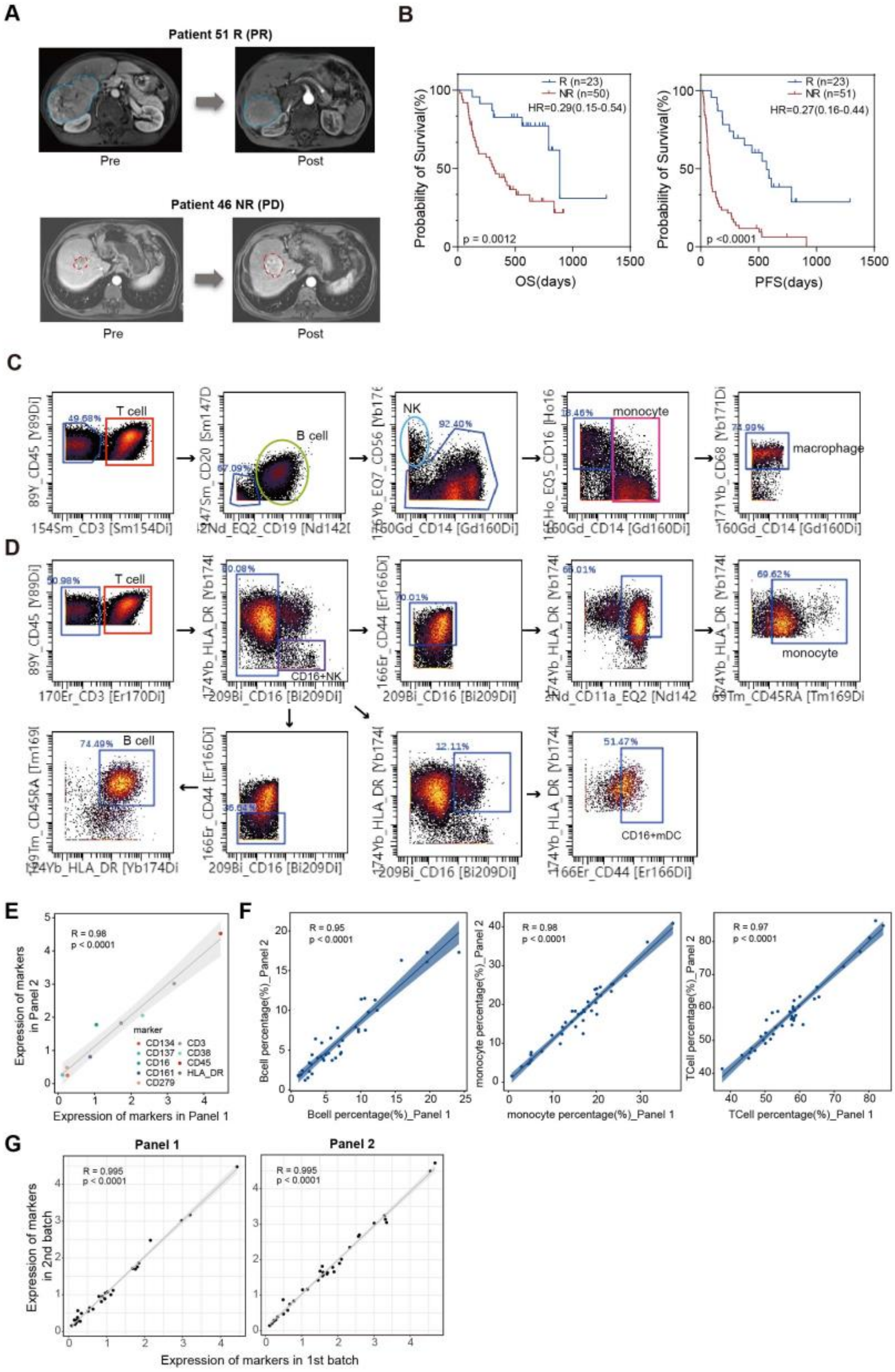

A
Patient 51 R (PR)
Pre
Post
Patient 46 NR (PD)
Pre
Post
B
R (n=23)
NR (n=50)
HR=0.29(0.15-0.54)
p = 0.0012
Probability of Survival(%)
OS(days)
R (n=23)
NR (n=51)
HR=0.27(0.16-0.44)
p <0.0001
PFS(days)
C
T cell
B cell
NK
monocyte
macrophage
D
T cell
CD16+NK
monocyte
B cell
CD16+mDC
E
R = 0.98
p < 0.0001
Expression of markers in Panel 2
Expression of markers in Panel 1
marker
CD134 CD3 CD137 CD38 CD16 CD45 CD161 HLA_DR CD279
F
R = 0.95
p < 0.0001
Bcell percentage(%)_Panel 2
Bcell percentage(%)_Panel 1
R = 0.98
p < 0.0001
monocyte percentage(%)_Panel 2
monocyte percentage(%)_Panel 1
R = 0.97
p < 0.0001
TCell percentage(%)_Panel 2
TCell percentage(%)_Panel 1
G
Panel 1
Panel 2
R = 0.995
p < 0.0001
Expression of markers in 2nd batch
Expression of markers in 1st batch

Figure S2

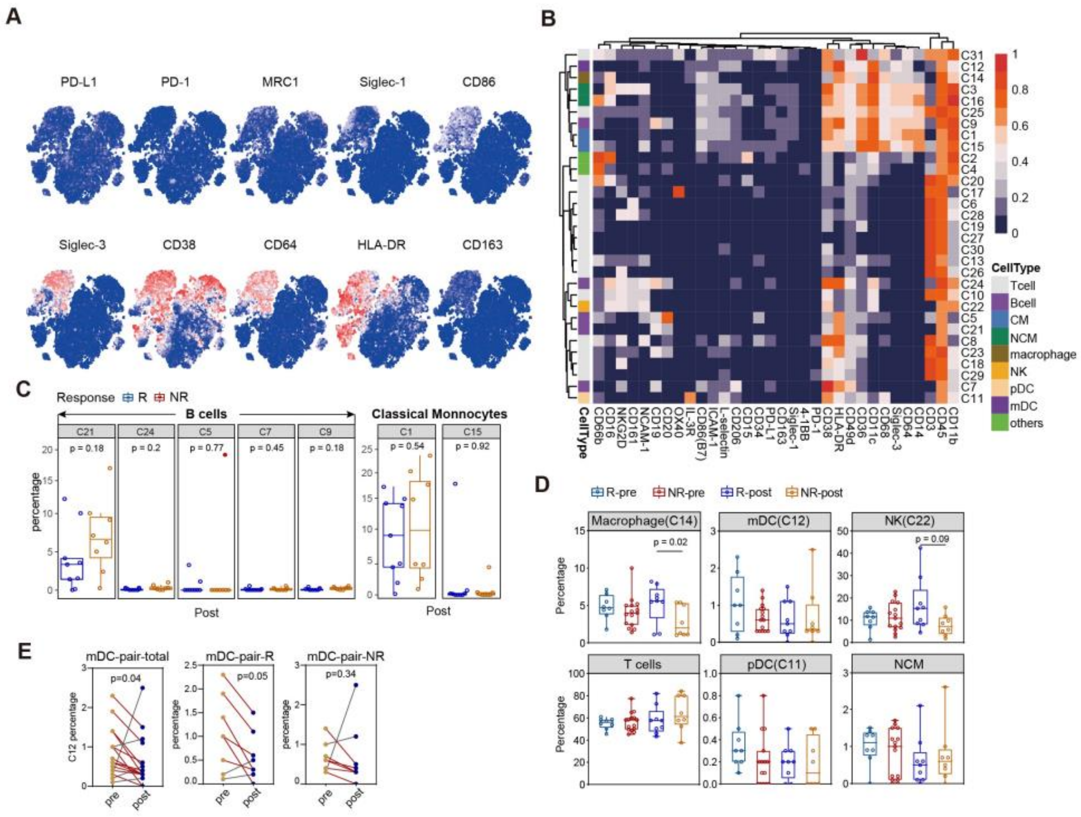
A
PD-L1
PD-1
MRC1
Siglec-1
CD86
Siglec-3
CD38
CD64
HLA-DR
CD163
B
CellType
Tcell
Bcell
CM
NCM
macrophage
NK
pDC
mDC
others
C
Response R NR
B cells
Classical Monnocytes
percentage
Post
D
R-pre NR-pre R-post NR-post
Macrophage(C14)
mDC(C12)
NK(C22)
T cells
pDC(C11)
NCM
Percentage
E
mDC-pair-total
mDC-pair-R
mDC-pair-NR
C12 percentage
percentage
pre
post

Figure S3

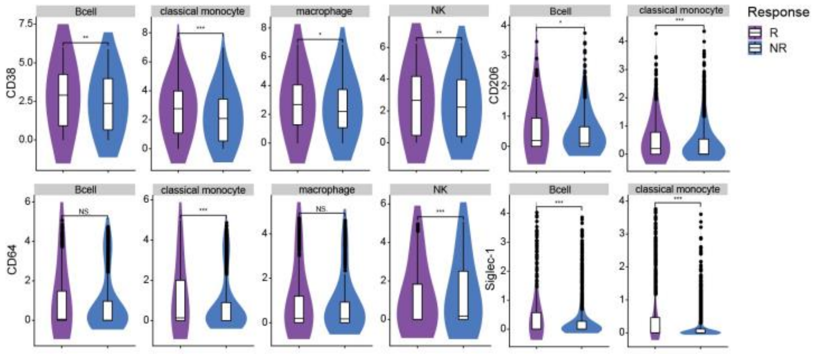
Bcell
classical monocyte
macrophage
NK
Bcell
classical monocyte
Response
R
NR
CD38
CD206
CD64
Siglec-1
NS
NS.

Figure S4

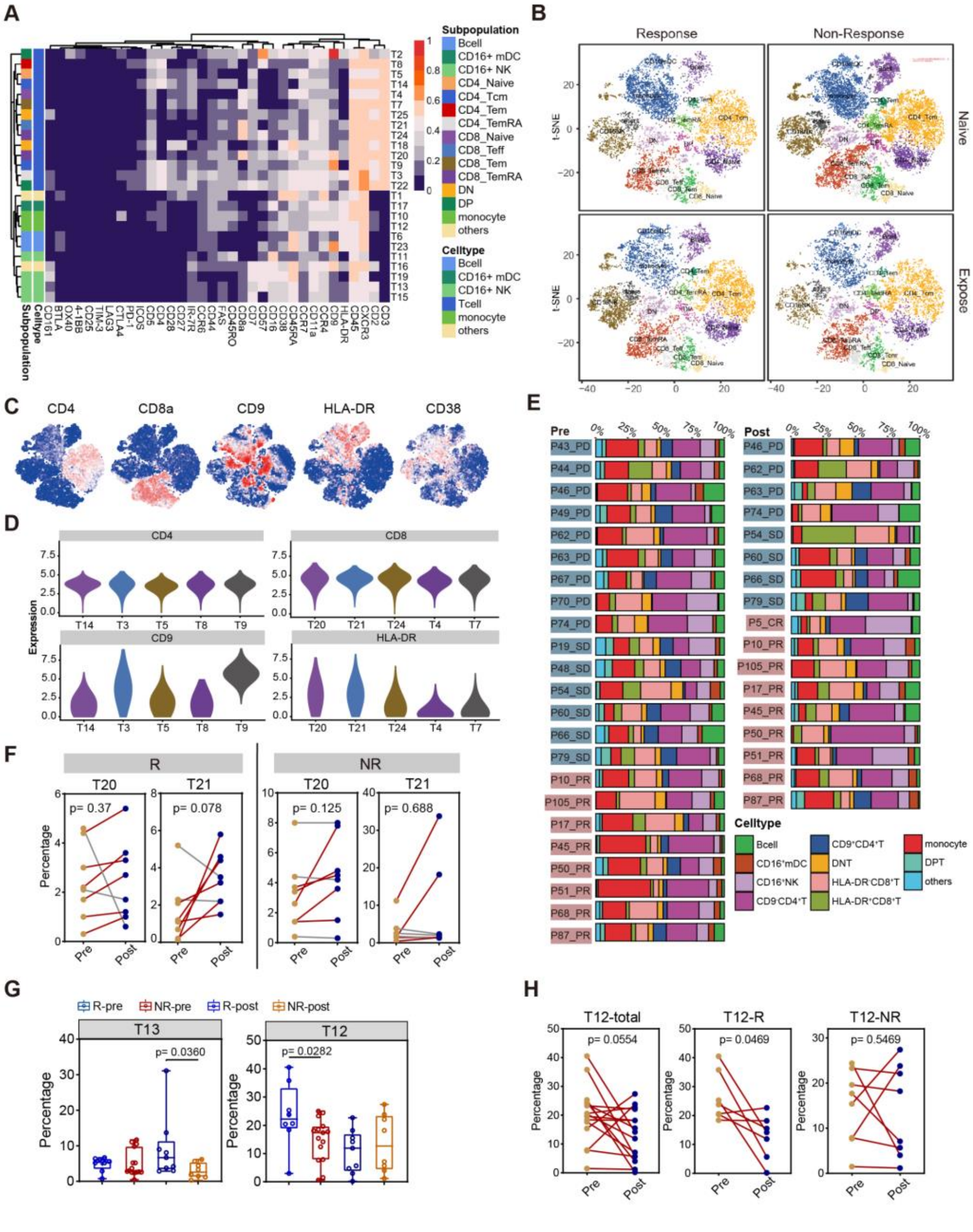
A
Subpopulation
Bcell
CD16+ mDC
CD16+ NK
CD4_Naive
CD4_Tcm
CD4_Tem
CD4_TemRA
CD8_Naive
CD8_Teff
CD8_Tem
CD8_TemRA
DN
DP
monocyte
others
Celltype
Bcell
CD16+ mDC
CD16+ NK
Tcell
monocyte
others
Subpopulation
Celltype
B
Response
Non-Response
Naive
Expose
t-SNE
C
CD4
CD8a
CD9
HLA-DR
CD38
D
CD4
CD8
CD9
HLA-DR
Expression
E
Pre
Post
Celltype
Bcell
CD16⁺mDC
CD16⁺NK
CD9⁻CD4⁺T
CD9⁺CD4⁺T
DNT
HLA-DR⁻CD8⁺T
HLA-DR⁺CD8⁺T
monocyte
DPT
others
F
R
NR
T20
T21
p= 0.37
p= 0.078
p= 0.125
p= 0.688
Percentage
Pre
Post
G
R-pre
NR-pre
R-post
NR-post
T13
T12
p= 0.0360
p= 0.0282
Percentage
H
T12-total
T12-R
T12-NR
p= 0.0554
p= 0.0469
p= 0.5469
Percentage
Pre
Post

Figure S5

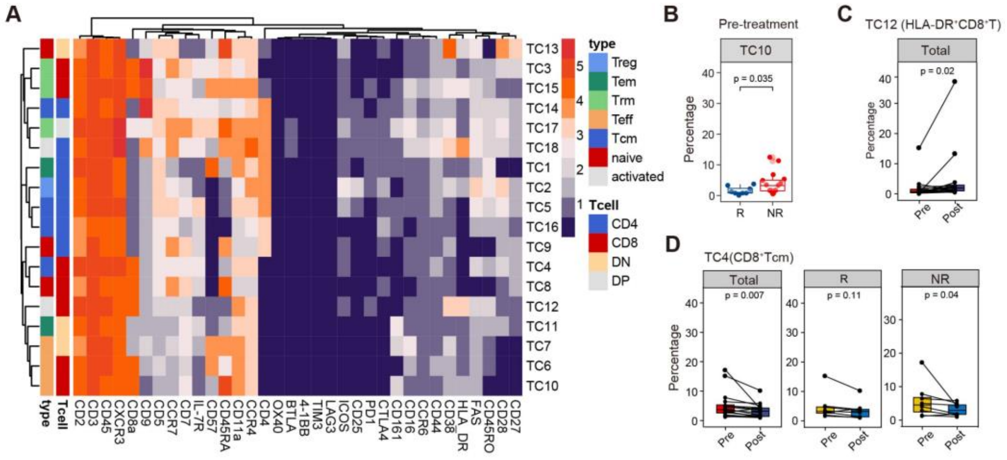

A
B
C
D
type
Treg
Tem
Trm
Teff
Tcm
naive
activated
Tcell
CD4
CD8
DN
DP
TC13
TC3
TC15
TC14
TC17
TC18
TC1
TC2
TC5
TC16
TC9
TC4
TC8
TC12
TC11
TC7
TC6
TC10
CD2
CD3
CD45
CXCR3
CD8a
CD9
CD5
CCR7
CD7
IL-7R
CD57
CD45RA
CD11a
CCR4
CD4
OX40
BTLA
4-1BB
TIM3
LAG3
ICOS
CD25
PD1
CTLA4
CD161
CD16
CCR6
CD44
CD38
HLA_DR
FAS
CD45RO
CD28
CD27
Pre-treatment
TC10
p = 0.035
Percentage
R
NR
TC12 (HLA-DR+CD8+T)
Total
p = 0.02
Pre
Post
TC4(CD8+Tcm)
p = 0.007
p = 0.11
p = 0.04

Figure S6

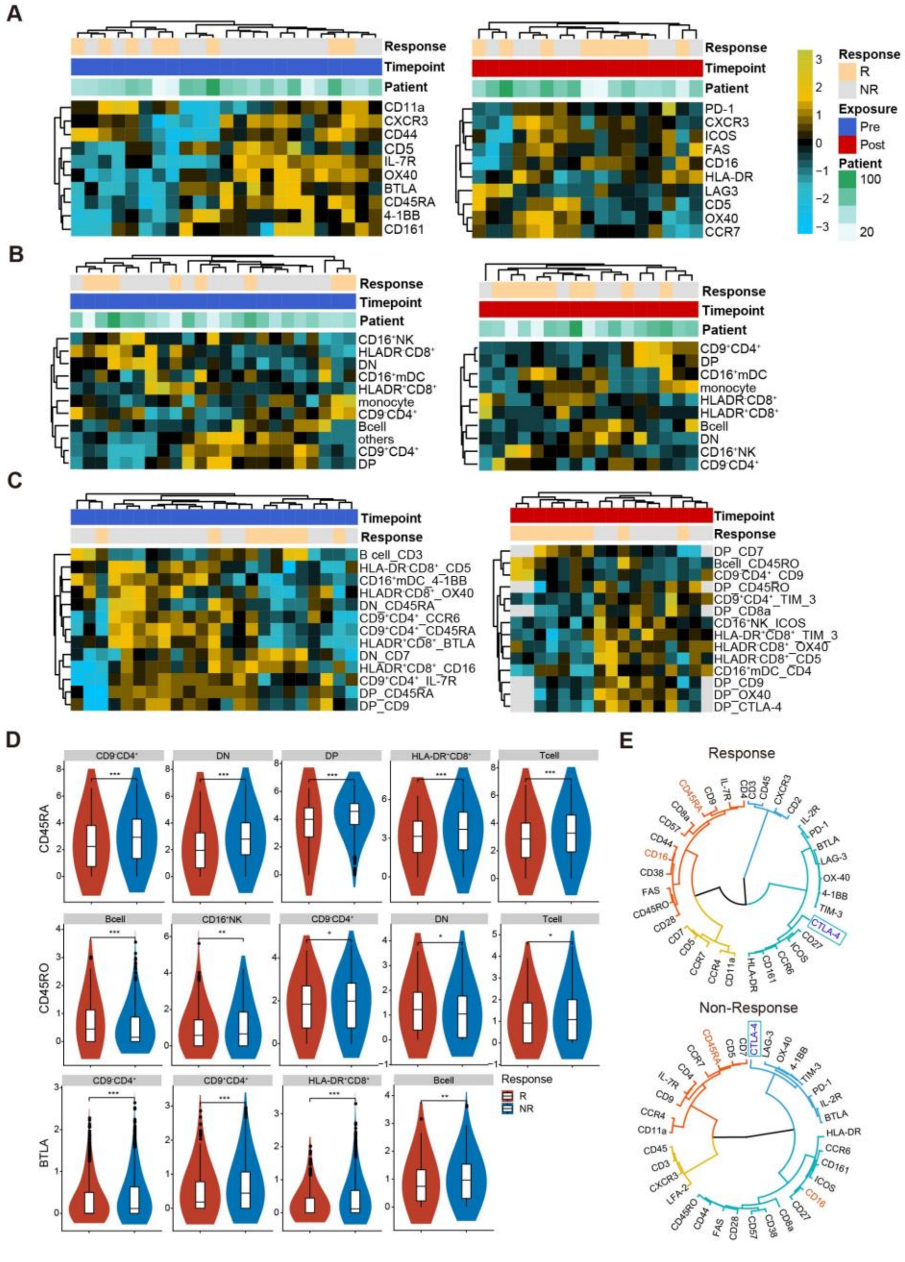
A
Response
Timepoint
Patient
CD11a
CXCR3
CD44
CD5
IL-7R
OX40
BTLA
CD45RA
4-1BB
CD161
PD-1
ICOS
FAS
CD16
HLA-DR
LAG3
CCR7
R
NR
Exposure
Pre
Post
100
20
B
CD16⁺NK
DN
CD16⁺mDC
monocyte
Bcell
others
DP
C
B cell_CD3
D
CD45RA
CD45RO
BTLA
DN
DP
Tcell
Bcell
E
Response
Non-Response
CTLA-4

Figure S7

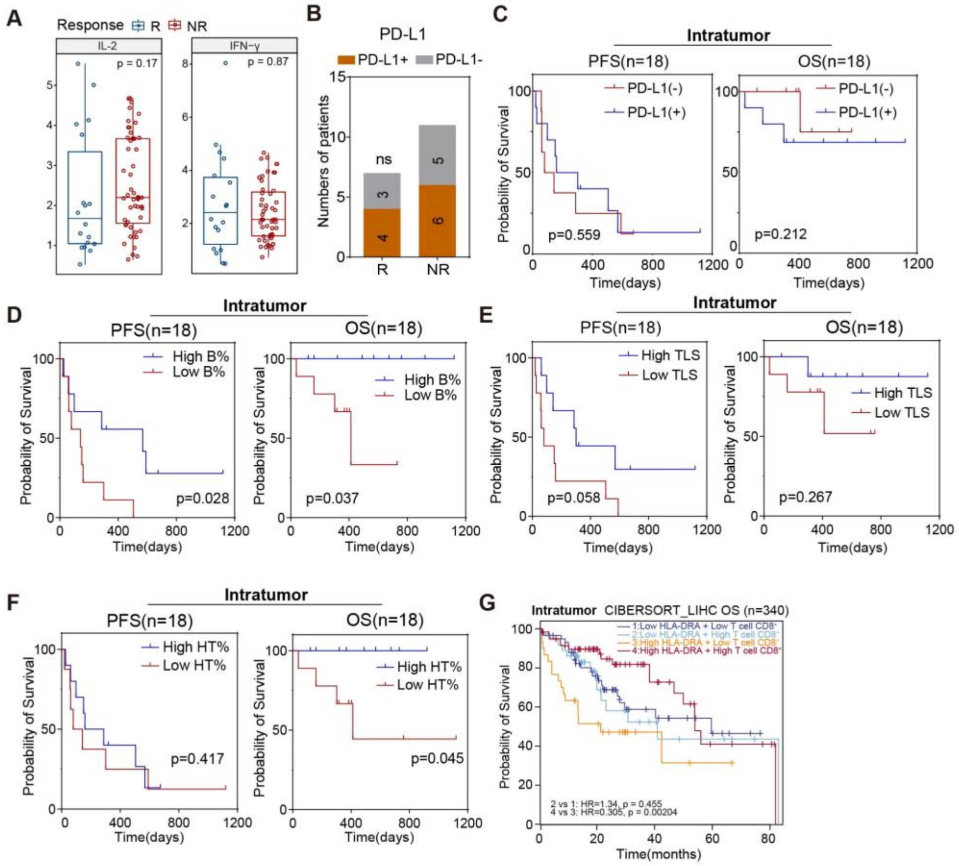

A
Response R NR
IL-2
p = 0.17
IFN-γ
p = 0.87
B
PD-L1
PD-L1+ PD-L1-
Numbers of patients
ns
R
NR
C
Intratumor
PFS(n=18)
OS(n=18)
PD-L1(-)
PD-L1(+)
Probability of Survival
p=0.559
p=0.212
Time(days)
D
Intratumor
PFS(n=18)
OS(n=18)
High B%
Low B%
p=0.028
p=0.037
E
Intratumor
PFS(n=18)
OS(n=18)
High TLS
Low TLS
p=0.058
p=0.267
F
Intratumor
PFS(n=18)
OS(n=18)
High HT%
Low HT%
p=0.417
p=0.045
G
Intratumor CIBERSORT_LIHC OS (n=340)
2 vs 1: HR=1.34, p = 0.455
4 vs 3: HR=0.305, p = 0.00204
Time(months)

Figure S8

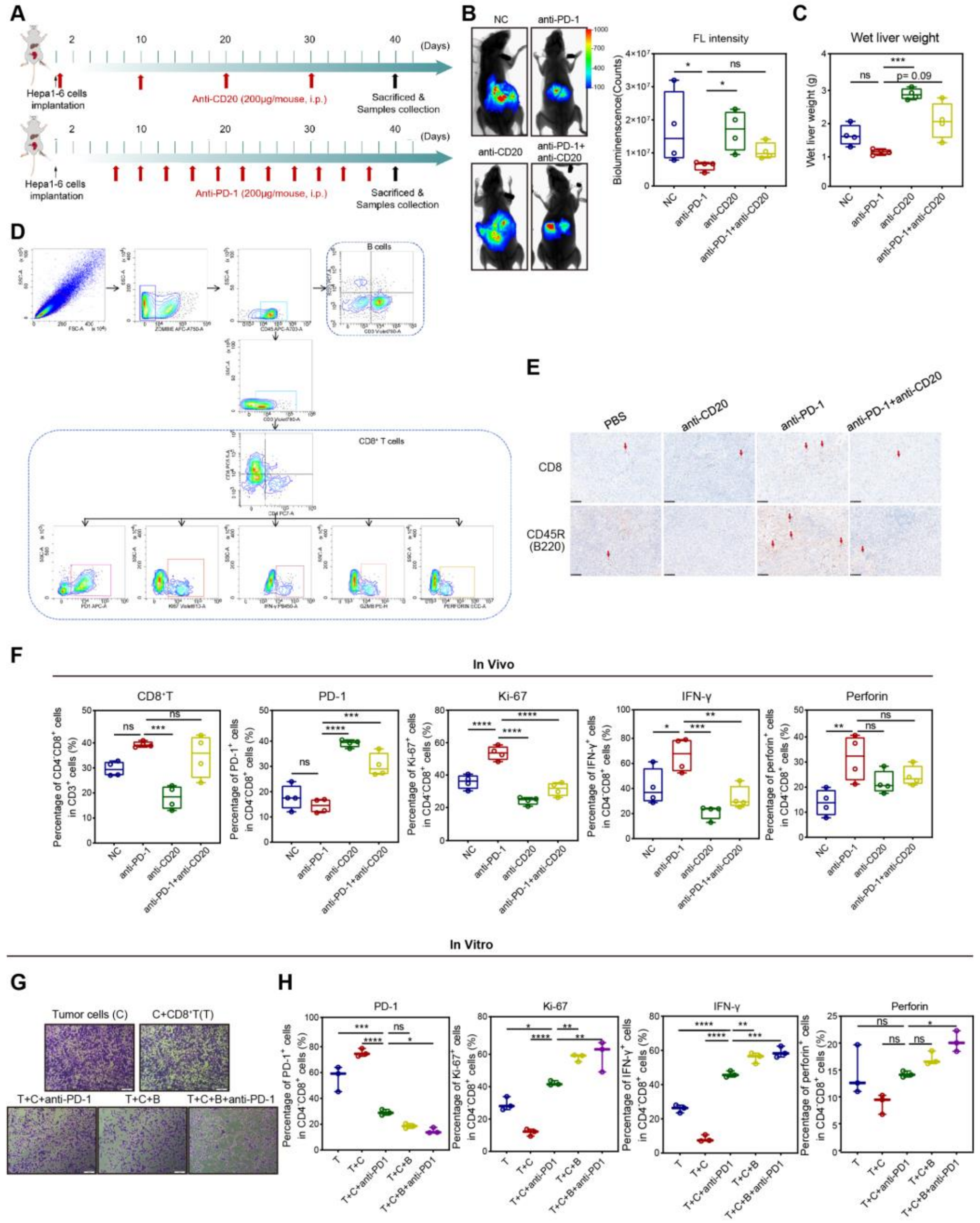
A
Hepa1-6 cells implantation
Anti-CD20 (200μg/mouse, i.p.)
Sacrificed & Samples collection
(Days)
Anti-PD-1 (200μg/mouse, i.p.)
B
NC
anti-PD-1
anti-CD20
anti-PD-1+ anti-CD20
FL intensity
Bioluminescence(Counts)
C
Wet liver weight
Wet liver weight (g)
p= 0.09
D
B cells
CD8⁺ T cells
E
PBS
anti-CD20
anti-PD-1
anti-PD-1+anti-CD20
CD8
CD45R (B220)
F
In Vivo
CD8⁺T
PD-1
Ki-67
IFN-γ
Perforin
In Vitro
G
Tumor cells (C)
C+CD8⁺T(T)
T+C+anti-PD-1
T+C+B
T+C+B+anti-PD-1
H
PD-1
Ki-67
IFN-γ
Perforin
