# Supplementary material for: Deciphering the Multifaceted Immune Landscape of Unresectable Primary Liver Cancer to Predict Immunotherapy Response
Source: Adv Sci (Weinh). 2024 Oct 28;11(47):2309631. doi: 10.1002/advs.202309631 (PMC11653612; doi:10.1002/advs.202309631)
Supplement: Supplementary file 2 — Supporting Information [file ADVS-11-2309631-s002.zip › TableS1_Clinical characteristics of PLC patients in CyTOF.docx]

Table S1. Clinicopathologic Characteristics of ICI-treated Cohorts tested using CyTOF

|  | Discovery cohort n=24 | Validation cohort 1  n=16 | P value |
| --- | --- | --- | --- |
| Sex, n (%) |  |  | >0.99 |
| Male | 20(83.33%) | 13(81.25%) |  |
| Female | 4(16.67%) | 3(18.75%) |  |
| Age, years (%) |  |  | 0.26 |
| <50 | 8(33.33%) | 2(12.50%) |  |
| ≥50 | 16(66.67%) | 14(87.50%) |  |
| Histological type, n (%) |  |  | 0.21 |
| ICC | 2(8.33%) | 1(6.25%) |  |
| HCC | 22(91.67%) | 15(93.75%) |  |
| HBV infection, n (%) |  |  | 0.20 |
| Positive | 22(91.67%) | 12(75.00%) |  |
| Negative | 2(8.33%) | 4(25.00%) |  |
| T Stage, n (%) |  |  | 0.17 |
| T2 | 2(8.33%） | 3(18.75%) |  |
| T3 | 18(75%.00) | 13(81.25%) |  |
| T4 | 4(16.67%) | 0 |  |
| N Stage, n (%) |  |  | <0.05 |
| N0 | 10(41.67%) | 14(87.50%) |  |
| N1 | 12(50.00%) | 2(12.50%) |  |
| NA | 2(8.33%) | 0 |  |
| M Stage, n (%) |  |  | 0.12 |
| M0 | 15(62.50%) | 6(37.50%) |  |
| M1 | 9(37.50%) | 10(62.50%) |  |
| BCLC Staging, n (%) |  |  | 0.23 |
| B | 3(12.50%) | 0 |  |
| C | 20(83.33%) | 16(100.00%) |  |
| NA | 1(4.17%) | 0 |  |
| Child-Pugh Score, n (%) |  |  | 0.34 |
| A | 20(83.34%) | 13(81.25%) |  |
| B | 2(8.33%) | 3(18.75%) |  |
| NA | 2(8.33%) | 0 |  |
| AFP, n (%) |  |  | 0.60 |
| <400 (μg/L) | 10(58.33%) | 8(50.00%) |  |
| ≥400 (μg/L) | 14(41.67%) | 8(50.00%) |  |
| CRP, n (%) |  |  | 0.08 |
| <1mg/dl | 2(8.34%) | 3(18.75%) |  |
| ≥1mg/dl | 11(45.83%) | 11(68.75%) |  |
| NA | 11(45.83%) | 2(12.50%) |  |
| Treatment, n (%) |  |  | >0.99 |
| ICIs | 9(37.50%) | 6(37.50%) |  |
| ICIs+Lenvatinib | 15(62.5%) | 10(62.5%) |  |
| Treatment line, n (%) |  |  | 0.08 |
| First line | 24(100.00%) | 14(87.50%) |  |
| Second line | 0 | 2(12.50%) |  |
| Best Response^a^, n (%) |  |  | <0.05 |
| CR | 1(4.17%) | 1(6.25%) |  |
| PR | 8(33.33%) | 5(31.25%) |  |
| SD | 5(20.83%) | 9(56.25%) |  |
| PD | 10(41.67%) | 1(6.25%) |  |

**Footnotes:** HBV, hepatitis B virus; BCLC Staging, Barcelona clinic liver cancer staging; CR, complete response; PR, partial response; SD, stable disease; PD, progressive disease; PFS, progression-free survival; OS, overall survival; CRP, c-reaction protein.

See also Figure S1 and Tables S1 and S2.

a. Patients were stratified into response groups based on RECIST 1.1 criteria. Patients with CR and PR were classified as responders, while patients with SD and PD were classified as non-responders.
